# Circulating microRNAs as biomarkers of disease activity and treatment response in Crohn’s disease patients receiving infliximab or vedolizumab: a longitudinal cohort study

**DOI:** 10.3389/fmed.2026.1923232

**Published:** 2026-08-13

**Authors:** Luisa Bertin, Lara Giraldi, Camilla Cavagna, Cristina Caranfil, Brigida Barberio, Romilda Cardin, Sonia Facchin, Chiara Carlotto, Milena Minotto, Edoardo Vincenzo Savarino, Fabiana Zingone

**Affiliations:** 1 Gastroenterology Unit, Department of Surgery, Oncology and Gastroenterology (DiSCOG), University of Padova, Padova, Italy; 2 Gastroenterology Unit, Azienda Ospedale-Università di Padova, Padova, Italy

**Keywords:** biomarker, Crohn’s disease, faecal calprotectin, infliximab, serum microRNA, treatment response, vedolizumab

## Abstract

**Background and aims:**

Validated biomarkers of biologic response in Crohn’s disease (CD) are lacking. We assessed whether serum microRNAs (miRNAs) track disease activity and predict outcomes during biologic induction in CD.

**Methods:**

Thirty-nine adults with active CD initiating infliximab (*n* = 20) or vedolizumab (*n* = 19) and 10 controls were studied at a single centre. Serum was sampled at the first and fourth infusions. Six target miRNAs were qPCR-quantified and normalised to the geometric mean of miR-93-5p and miR-425-5p. Harvey–Bradshaw Index, C-reactive protein, faecal calprotectin and Simple Endoscopic Score for CD were recorded at baseline, post-induction and 12 months. Four pre-specified analysis families were tested with false discovery rate (FDR) correction for multiple testing. This was a single-centre, exploratory, hypothesis-generating study.

**Results:**

miR-21-5p and miR-146b-5p were significantly reduced in CD versus controls (q < 0.05). Baseline miR-146b-5p inversely predicted post-induction CRP independently of baseline disease severity (partial r = −0.46, *p* = 0.010), supporting it as a candidate severity-independent predictor of biochemical response. Post-induction miR-424-5p strongly tracked concurrent inflammation (faecal calprotectin: Spearman *ρ* = +0.52, q = 0.004; CRP: ρ = +0.44, q = 0.030), these associations remained significant after adjustment for baseline disease severity, after leave-one-out removal of single patients, and under single-reference normalisation. An exploratory post-hoc analysis linked treatment-induced miR-106a-5p upregulation to penetrating (Montreal B3) disease (permutation *p* = 0.019).

**Conclusion:**

Serum miRNAs offer novel predictive (baseline miR-146b-5p and miR-106a-5p) and concurrent (post-induction miR-424-5p) biomarker signals during biologic induction in CD, providing information complementary to faecal calprotectin. Prospective independent validation is warranted before clinical translation.

## 1 Introduction

Crohn’s disease is a chronic, relapsing, transmural inflammatory disorder of the gastrointestinal tract whose management has been transformed over the past two decades by biologic agents targeting tumour necrosis factor-*α* (anti-TNF, including infliximab and adalimumab) and the gut-selective integrin α4β7 (vedolizumab) (1). Despite these advances, only 30–50% of patients achieve durable response to any single biologic, and 10–40% lose response over time (2). The empirical sequential approach to biologic selection that follows from this uncertainty contributes to substantial avoidable cost, prolonged active disease, and progression to fibrostenotic or penetrating complications (3, 4).

Non-invasive monitoring is central to contemporary IBD management, with the field steadily moving away from invasive assessment toward serum- and stool-based tools (5–7). Faecal calprotectin and C-reactive protein remain the dominant non-invasive biomarkers in current clinical practice (8). Faecal calprotectin in particular has demonstrated impressive sensitivity and specificity for active mucosal inflammation, but neither marker reliably predicts treatment response, distinguishes inflammatory from fibrostenotic disease behaviour, nor captures the deep-bowel-wall pathology characteristic of penetrating CD. Endoscopy with histological assessment remains the diagnostic gold standard but is invasive and inadequately reflects transmural disease (9).

MicroRNAs — small non-coding RNAs that regulate gene expression post-transcriptionally — have emerged as candidate non-invasive biomarkers because of their stability in blood, ease of quantification, and biological relevance to immune and barrier function (10). Multiple miRNAs have been implicated in IBD pathogenesis. Among them, miR-21-5p and miR-146b-5p regulate NF-κB signalling and have been reported as differentially expressed in IBD; miR-106a-5p targets interleukin-10 (IL-10) and modulates macrophage signalling through SIRPα; miR-424-5p has been characterised as a profibrotic effector in liver, lung, and oral submucosal fibrosis (11). However, there is still methodological heterogeneity in the field and the absence of independent validation as the principal barriers to clinical translation.

Two specific gaps motivated this study. First, longitudinal data linking pre-treatment miRNA levels to treatment outcomes in CD biologic patients are limited and largely confined to small exploratory cohorts. Second, although the relationship between circulating miRNAs and CD disease behaviour (and particularly the penetrating phenotype) has biological plausibility, it has been incompletely characterised in blood; we explored phenotype-stratified analyses in this study as exploratory rather than confirmatory. With these premises we conducted a longitudinal study of six pre-specified miRNAs in serum from CD patients receiving infliximab or vedolizumab induction therapy, sampled before and after induction, with three aims: to characterise miRNA dysregulation relative to healthy controls; to identify miRNAs whose pre-treatment levels or treatment-induced changes predict subsequent clinical, biochemical, or endoscopic outcomes; and to evaluate whether miRNAs add information beyond established non-invasive biomarkers for either disease-activity assessment or outcome prediction.

## 2 Materials and methods

### 2.1 Study design and patient enrolment

We conducted a prospective single-centre cohort study at the Gastroenterology Unit of Azienda Ospedale-Università di Padova (Padova, Italy). Consecutive eligible adults (≥ 18 years) had an established diagnosis of Crohn’s disease per ECCO criteria (12) and were starting biologic induction with infliximab (5 mg/kg at weeks 0, 2, 6) or vedolizumab (300 mg at weeks 0, 2, 6) per standard clinical practice (ie. moderate-to-severe disease activity) (13). The choice of biologic was at the discretion of the treating clinician; consistent with contemporary practice, anti-TNF was used predominantly in biologic-naïve patients while vedolizumab was used in patients with prior anti-TNF exposure or comorbidities limiting TNF inhibition. Healthy controls (*n* = 10), recruited from hospital staff and visitors with no history of IBD or other gastrointestinal disease, contributed a single serum sample to serve as the reference for ΔΔCt calculations. The HC pool had a median age of 33 years (60% female, median BMI 21.3 kg/m^2^, 10% current smokers), broadly similar to the patient cohort but not formally matched. The HC group was used as a reference for baseline CD-vs-HC contrasts only; the main within-patient analyses (paired change over induction, predictive associations with outcomes) do not depend on the HC reference.

The study was approved by the Ethical Committee of the Coordinating Centre (Padova University Hospital, Padova, Italy) on 25 May 2022 (Ref CESC 5370/AO/22). The study protocol conforms to the ethical guidelines of the 1975 Declaration of Helsinki.

### 2.2 Sample collection and miRNA quantification

Whole blood (10 mL) was collected in serum-separator tubes and allowed to clot at room temperature; serum was then separated by standard centrifugation within 2 h of collection and stored at −80 °C until processing. For miRNAs study, analysis was performed on serum samples from patients and healthy controls. Total RNA, including small RNAs, was extracted from 200 μL of serum using the miRNeasy Serum/Plasma Advanced Kit (Qiagen, Hilden, Germany) and eluted in 20 μL of RNase-free water. Extraction efficiency was monitored using synthetic spike-in controls (UniSp2, UniSp4, UniSp3). cDNA synthesis was performed using the miRCURY LNA RT Kit (Qiagen) according to the manufacturer’s instructions. Reverse transcription reactions contained 4 μL of RNA template and included UniSp6 as a control for reverse transcription efficiency. Relative miRNA expression of miR-21-5p, miR-146b-5p, miR-106a-5p, miR-362-3p, miR-376c-3p, miR-424-5p was assessed by RT-PCR using miRCURY LNA miRNA PCR Assays and Panels (Qiagen) on a PRISM 7900HT system (Applied Biosystems, Foster City, CA, USA). Each reaction contained SYBR Green Master Mix, PCR primer mix, and diluted cDNA. Cycling conditions were 95 °C for 2 min, followed by 40 cycles at 95 °C for 10 s and 56 °C for 60 s, with subsequent melting curve analysis. Relative miRNA expression levels were calculated using the comparative cycle threshold 2^-ΔΔCt^ method and expressed as fold change (FC). miR-93-5p and miR-425-5p were used as endogenous controls for normalization, both established in the serum miRNA literature as stable references and selected on the basis of low inter-sample variability across all samples in our panel. The stability of miR-93-5p and miR-425-5p was further confirmed using NormFinder and GeNorm-like approaches, showing low variability (M < 0.5) and optimal combined stability (Supplementary Table S1). Outlier exclusion further improved stability without affecting biological conclusions. For all statistical analyses these values were log2-transformed and are reported throughout as log2 fold-change (that is, log2 of the 2^(-ΔΔCt) fold change) relative to the healthy-control reference.

Healthy controls were used as the reference group. All laboratory procedures were performed following established methods and standardised protocols, with particular attention to minimizing inter-assay and intra-assay variability.

The six target miRNAs were selected *a priori* based on prior reports of dysregulation in IBD blood and tissue and on biological involvement in distinct regulatory pathways: NF-κB / Toll-like receptor signalling (miR-21-5p, miR-146b-5p), macrophage polarisation and Treg homeostasis (miR-106a-5p, miR-362-3p), and intestinal fibrosis / monocyte–myofibroblast differentiation (miR-376c-3p, miR-424-5p) (14). The specific evidence base motivating each miRNA’s inclusion is summarised in Supplementary Table S2.

### 2.3 Clinical and biochemical assessments

Demographic and disease characteristics were recorded at baseline, including age, sex, body mass index, smoking status, family history of IBD, disease duration, Montreal location (L1–L3 with L4 modifier) and behaviour (B1–B3) classification, perianal disease, prior surgical history, and prior pharmacological exposures. Clinical activity was assessed by the Harvey–Bradshaw Index (HBI). C-reactive protein (CRP, reported in mg/L; upper limit of normal in our laboratory 5 mg/L) and faecal calprotectin (FC, μg/g) were measured at standard hospital laboratories. Endoscopy with assessment of the Simple Endoscopic Score for Crohn’s Disease (SES-CD) was performed at baseline and at 12 months where clinically indicated.

Pre-specified outcome definitions were as follows. Clinical remission post-induction: HBI < 5 at the post-induction visit. Biochemical remission post-induction: FC < 250 μg/g at the post-induction visit. Treatment success at last follow-up was defined as the composite of (a) treatment continuation through the last recorded visit, OR (b) therapy discontinued because of sustained remission. Treatment failure was defined as therapy discontinuation due to inefficacy, loss of response, or worsening of disease.

miRNAs were measured only at the two protocol timepoints (two serum collections per patient, pre- and post-induction, rather than serial sampling; “longitudinal” throughout refers to this paired pre/post design and to the use of these baseline and post-induction values as predictors of later outcomes, not to repeated measurement at 12 months) (pre- and post-induction); we used baseline and post-induction miRNA values as predictors of 12-month clinical and endoscopic outcomes, not measurements at 12 months. The 12-month SES-CD score was available in 77% of patients (90% INFX, 63% VDZ); the lower VDZ availability reflects therapy discontinuation before the 12-month endoscopy. Analyses involving 12-month outcomes used complete-case data; multiple imputation was not performed because of the modest sample size and the clinically driven nature of the missingness, which we have flagged as a limitation. Among the 39 patients, HBI was available in 39 at baseline, 39 post-induction and 35 at 12 months; CRP in 39, 39 and 37; faecal calprotectin in 39, 39 and 38; and SES-CD in 30 at baseline and 30 at 12 months.

### 2.4 Statistical analysis

Analyses were performed in R version 4.5.0 (R Core Team, 2025) and Python 3.12 (scipy 1.13, scikit-learn 1.4). Continuous variables are summarised as median (interquartile range, IQR) and compared by Mann–Whitney U (between groups) or Wilcoxon signed-rank (paired pre/post). Categorical variables are summarised as *n* (%) and compared by Fisher’s exact test. Spearman rank correlation was used for continuous–continuous associations. Each headline correlation was re-computed with each single observation removed in turn (leave-one-out single-observation sensitivity), with the range of resulting Spearman *ρ* and any qualitative direction or significance changes reported. No outlier trimming or winsorisation was applied; non-parametric methods were used throughout precisely to avoid the need for distributional assumptions or outlier handling.

Four pre-specified analysis families were tested: (F1) baseline HC vs. CD; (F2) paired pre- vs. post-induction change within drug arm; (F3) INFX vs. VDZ; (F4) miRNA × outcome predictive associations. Multiple testing was controlled within each family using the Benjamini–Hochberg false discovery rate (FDR) at q < 0.05. FDR correction was applied within each pre-specified family but not across families; in addition to the pre-specified families, several exploratory contrasts (drug-arm subgroups, the Montreal B3 phenotype contrast, and 12-month outcomes) were examined, so that the predictive (F4) family alone comprised a pre-specified grid of 648 miRNA × outcome combinations (six miRNAs × three predictor timepoints × twelve outcomes × three cohort strata), at which scale approximately 32 nominally significant associations (about 5%) would be expected by chance in the absence of correction. Given the sample size (*n* = 39), findings that did not survive FDR correction are reported throughout as candidate, hypothesis-generating signals rather than as confirmed associations. Partial Spearman correlations adjusted for baseline severity (HBI, SES-CD, CRP, FC). Drug-stratified analyses are exploratory because n = 39 is too small for formal interaction tests. No formal *a priori* sample-size calculation was performed: the cohort comprised all eligible patients enrolled at the single participating centre during the study window, and the study was designed as an exploratory, hypothesis-generating biomarker analysis rather than to test a pre-specified effect size. A post-hoc consideration indicates that, for a two-sided Spearman correlation at *α* = 0.05 with *n* = 39, the analysis had approximately 80% power to detect a moderate association (|*ρ*| ≈ 0.43); smaller effects, and most drug-stratified contrasts, were correspondingly underpowered, which is why such findings are reported as candidate and hypothesis-generating. A fixed random seed of 42 was used for all stochastic procedures (bootstrap resampling, permutation testing, sensitivity sampling). Stability of the principal LOO-CV AUC and bootstrap CI was confirmed by re-running the headline VDZ post-induction miR-146b-5p × treatment-success analysis under five alternative seeds (1, 7, 100, 2024, 42); the LOO-CV AUC point estimate is deterministic and identical across seeds (0.79), and bootstrap 95% CI bounds varied by less than 0.03. Specifically, Benjamini-Hochberg was applied across the six panel miRNAs within each cohort by predictor-timepoint by outcome cell. When the correction is instead applied across the entire predictive family, only the post-induction miR-424-5p by faecal calprotectin association remains close to the threshold (q = 0.054; Supplementary Table S3), so all predictive and concurrent associations are interpreted throughout as robust candidate, hypothesis-generating signals, characterised by effect size, confidence interval and stability under sensitivity analysis rather than as grid-wide confirmed findings.

We performed one post-hoc phenotype-stratified contrast on the strongest *a priori* biological hypothesis (Montreal B3 vs. non-B3 contrast for treatment-induced miR-106a-5p change, motivated by the IL-10/SIRPα biology); this is reported in section 3.7 with a permutation-based *p*-value (50,000 shuffles).

Discriminative performance was assessed by leave-one-patient-out cross-validated AUC, with both observations from a given patient held out together, and 1,500 patient-clustered bootstrap resamples for 95% confidence intervals. Active disease at any patient-timepoint was operationally defined for cross-sectional discrimination as HBI ≥ 5, with faecal calprotectin and miRNAs evaluated against this FC-independent reference label to avoid tautological circularity. Validated miRNA targets were retrieved from miRTarBase v9.0 (release 2022) and intersected with curated IBD-pathway gene lists; pathway enrichment is interpreted as a relative pattern rather than absolute biological assignment. Statistical significance was defined as q < 0.05 for FDR-controlled tests within pre-specified families and as *p* < 0.05 for confirmatory comparisons.

A MIQE checklist for qPCR reporting is provided as Supplementary material. Reporting follows the STROBE recommendations for observational cohort studies. The primary analyses were the four pre-specified families F1 to F4 with false discovery rate control; the drug-arm subgroup contrasts, the Montreal B3 phenotype comparison, the 12-month endoscopic outcomes and the cross-sectional activity discrimination were secondary and exploratory.

## 3 Results

### 3.1 Patient cohort and clinical context

We enrolled 39 patients with Crohn’s disease — 20 starting infliximab and 19 starting vedolizumab — together with 10 healthy controls. Baseline characteristics are summarised in Table 1.

**Table 1:** Baseline characteristics of the study population (HC, INFX, VDZ, all patients), stratified by treatment arm with INFX-vs-VDZ comparison.

Variable	Healthy controls (*n* = 10)	All patients (*n* = 39)	Infliximab (*n* = 20)	Vedolizumab (*n* = 19)	*p* (INFX vs. VDZ)
Demographics					
Age, years	33.1 (22.3–42.7)	37.2 (24.9–53.0)	32.9 (24.7–46.1)	41.8 (28.3–54.2)	0.267
Sex, female, *n* (%)	6 (60%)	16 (41%)	11 (55%)	5 (26%)	0.105
BMI, kg/m^2^	21.3 (20.2–23.1)	23.7 (21.1–25.1)	22.5 (20.5–24.1)	24.1 (22.3–26.4)	0.049
Current smoker, *n* (%)	1 (10%)	5 (13%)	1 (5%)	4 (21%)	0.182
Disease characteristics					
Disease duration, years	—	5.9 (1.5–15.8)	2.0 (0.8–8.3)	15.8 (4.6–21.4)	<0.001
Family history of IBD, *n* (%)	—	7 (18%)	3 (15%)	4 (21%)	0.695
Extra-intestinal manifestations, *n* (%)	—	3 (8%)	1 (5%)	2 (11%)	0.605
Montreal classification					
L1 (ileal), *n* (%)	—	7 (18%)	5 (25%)	2 (11%)	
L2 (colonic), *n* (%)	—	9 (23%)	4 (20%)	5 (26%)	
L3 (ileocolonic), *n* (%)	—	23 (59%)	11 (55%)	12 (63%)	
L4 modifier (upper GI), *n* (%)	—	1 (3%)	1 (5%)	0 (0%)	
B1 (inflammatory), *n* (%)	—	12 (31%)	5 (25%)	7 (37%)	
B2 (stricturing), *n* (%)	—	8 (21%)	4 (20%)	4 (21%)	
B3 (penetrating), n (%)	—	19 (49%)	11 (55%)	8 (42%)	
Perianal disease, *n* (%)	—	12 (31%)	7 (35%)	5 (26%)	0.731
Prior history					
Prior IBD luminal surgery, *n* (%)	—	15 (38%)	8 (40%)	7 (37%)	1.000
Prior perianal surgery, *n* (%)	—	10 (26%)	7 (35%)	3 (16%)	0.273
Prior systemic steroids, *n* (%)	—	22 (56%)	12 (60%)	10 (53%)	0.751
Prior azathioprine, *n* (%)	—	12 (31%)	2 (10%)	10 (53%)	0.006
Prior anti-TNF biologic exposure, *n* (%)	—	22 (56%)	7 (35%)	15 (79%)	0.010
Baseline disease activity					
Harvey-Bradshaw Index, score	—	12.0 (10.0–14.0)	12.0 (10.8–14.2)	12.0 (9.5–14.0)	0.600
HBI ≥ 5 (clinically active), *n* (%)	—	38 (97%)	20 (100%)	18 (95%)	0.487
SES-CD score	—	7.5 (5.0–11.8)	7.0 (4.8–10.0)	9.5 (5.2–18.0)	0.359
CRP, mg/L	—	2 (0–38)	0.5 (0.2–30.5)	15 (2–78)	0.002
CRP > 5 mg/L, *n* (%)	—	18 (46%)	6 (30%)	12 (63%)	0.056
Faecal calprotectin, μg/g	—	633 (356–1,252)	764 (579–1,377)	406 (210–944)	0.021
FC ≥ 250 μg/g, *n* (%)	—	32 (82%)	18 (90%)	14 (74%)	0.235

Continuous variables are presented as median (IQR); categorical as *n* (%). Statistical tests: Mann–Whitney U for continuous, Fisher’s exact for binary. The “% with elevated CRP” and “% with elevated faecal calprotectin” rows are included to provide a clearer picture of baseline activity, given the bimodal distribution of CRP in CD (many patients have normal CRP despite clinically and endoscopically active disease, particularly in ileal-predominant phenotypes).

The cohort was representative of CD patients in a tertiary IBD practice initiating biologic therapy: median age 37.2 years (IQR 24.9–53.0), 41% female, with predominantly ileocolonic disease (Montreal L3 in 59%) and a high proportion of penetrating-behaviour disease (B3 in 49%, with perianal disease in 31%). Disease was clinically active in nearly all patients at baseline: 97% had a Harvey-Bradshaw Index ≥ 5, with median HBI of 12 (IQR 10–14). Endoscopic activity was moderate-to-severe (median SES-CD 7.5, IQR 5.0–11.8). Faecal calprotectin was elevated (≥ 250 μg/g) in 82% of patients (median 633 μg/g, IQR 356–1,252). C-reactive protein was elevated (> 5 mg/L) in 46%.

The two biologic arms differed in characteristics that reflect contemporary clinical positioning rather than randomisation. Patients starting vedolizumab had longer disease duration (median 15.8 vs. 2.0 years, *p* < 0.001), higher prior anti-TNF exposure (79% vs. 35%, *p* = 0.010), higher prior azathioprine exposure (53% vs. 10%, *p* = 0.006), higher baseline CRP (median 15.0 vs. 0.5 mg/L, *p* = 0.002), and higher baseline body-mass index (24.1 vs. 22.5 kg/m^2^, *p* = 0.049). Patients starting infliximab had higher baseline faecal calprotectin (764 vs. 406 μg/g, *p* = 0.021), reflecting more biologic-naïve disease at biologic initiation.

Treatment outcomes are summarised in Table 2.

**Table 2:** Treatment outcomes at post-induction (before the fourth infusion, approximately week 14) and at last follow-up/12 months, with each component of the treatment-success composite (continuation through last follow-up; therapy stopped due to sustained remission) tabulated separately.

Variable	Infliximab (*n* = 20)	Vedolizumab (*n* = 19)	All patients (*n* = 39)	*p* (INFX vs. VDZ)
Post-induction (4th infusion)				
HBI score	1.0 (0.0–3.2)	1.0 (1.0–3.0)	1.0 (0.0–3.0)	0.615
Clinical remission (HBI < 5), *n* (%)	17 (85%)	16 (84%)	33 (85%)	1.000
CRP, mg/L	2.0 (1.8–5.0)	5.0 (3.0–24.5)	4.0 (2.0–7.0)	0.021
CRP > 5 mg/L, *n* (%)	4 (20%)	8 (42%)	12 (31%)	0.176
Faecal calprotectin, μg/g	68 (21–361)	70 (31–209)	70 (28–238)	0.789
FC < 250 μg/g (biochemical remission), *n* (%)	14 (70%)	15 (79%)	29 (74%)	0.716
Long-term follow-up (12 months)				
12-month SES-CD score available, *n* (%)	18 (90%)	12 (63%)	30 (77%)	0.065
SES-CD at 12 months	2.0 (0.0–6.8)	2.0 (1.8–3.0)	2.0 (0.0–4.5)	0.696
Treatment success at last follow-up, *n* (%)	11/15 (73%)	8/17 (47%)	19/32 (59%)	0.166

The contrast between baseline values (Table 1) and post-induction values (Table 2) demonstrates substantial response: Faecal calprotectin elevation dropped from 82 to 26%; clinical remission was achieved by 85% of patients; biochemical remission by 74%. The apparent stability of the median CRP (2.0 mg/L at baseline → 4.0 mg/L post-induction) reflects the bimodal CRP distribution and the dramatic narrowing of the upper interquartile range (Q3 dropped from 37.5 to 7.0 mg/L); the proportion of patients with elevated CRP (> 5 mg/L) decreased from 46 to 31%, confirming clinical response.

Clinical remission post-induction was achieved by 85% of patients overall (INFX 85%, VDZ 84%). Biochemical remission (FC < 250 μg/g) was achieved by 74% (INFX 70%, VDZ 79%). At last follow-up, treatment success was recorded in 11/15 (73%) of infliximab-treated and 8/17 (47%) of vedolizumab-treated patients with a determinable outcome; the apparent difference (risk difference +26.3%, 95% CI − 6.3% to +58.9%; odds ratio 3.09, 95% CI 0.70 to 13.71; Fisher’s *p* = 0.166) is non-significant with a confidence interval crossing both zero and unity, consistent with the underpowered nature of the between-arm comparison. The qualitative direction is consistent with the more refractory disease profile of the vedolizumab arm (longer disease duration, prior anti-TNF failure). A 12-month SES-CD score was available for 77% of patients (90% INFX, 63% VDZ; the lower VDZ proportion reflects therapy discontinuation prior to the planned endoscopy). 22 patients discontinued therapy during follow-up: of the 22 discontinuations, 7 (3 INFX, 4 VDZ) occurred within the 12-month follow-up window and the remaining 14 occurred during extended follow-up (median time to discontinuation 16 months, range 5 weeks to 47 months); 17 patients (9 INFX, 8 VDZ) remained on therapy at last recorded visit.

### 3.2 miRNA dysregulation in CD versus healthy controls

**Figure 1:**
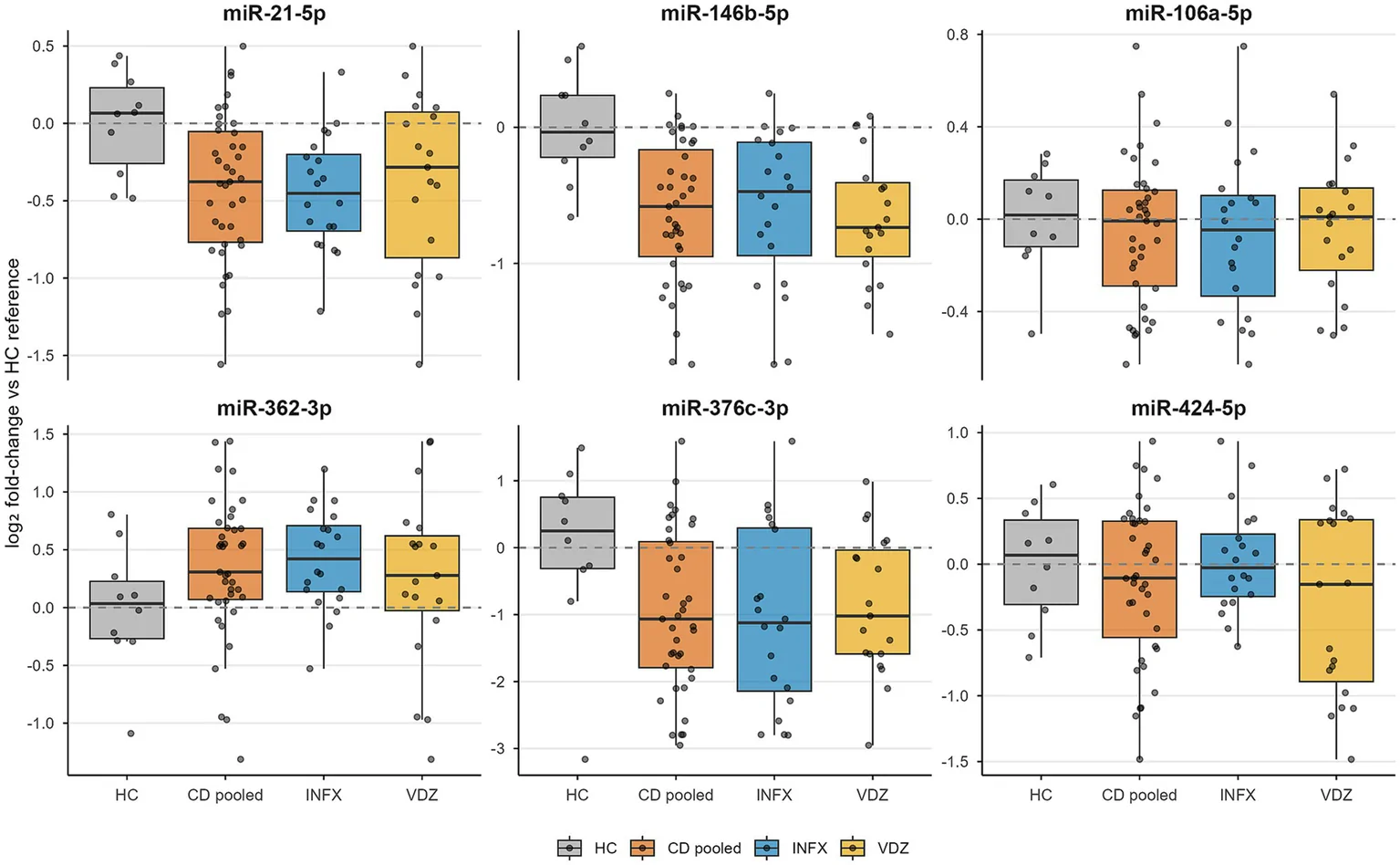
Baseline serum levels of the six panel miRNAs in healthy controls (HC, *n* = 10) versus Crohn’s disease patients shown as the pooled CD cohort (*n* = 39) and as INFX (*n* = 20) and VDZ (*n* = 19) subgroups. Values are log₂ fold-change relative to the pooled HC reference (Cq normalised to the geometric mean of miR-93-5p and miR-425-5p). Boxes show median and IQR with raw data points overlaid. Dashed line at zero marks the HC reference level. Mann–Whitney U-test with Benjamini–Hochberg FDR within each comparison family. Two of the six miRNAs were reduced in CD vs. HC after FDR correction: miR-21-5p (*q* = 0.035) and miR-146b-5p (*q* = 0.012). The same two miRNAs were also reduced within the INFX and VDZ subgroups (miR-146b-5p in both arms; miR-21-5p in INFX only). The other four miRNAs did not differ significantly between HC and CD at baseline.

At baseline, two of the six miRNAs were significantly reduced in CD serum compared with HC after FDR correction: miR-146b-5p (pooled q = 0.012; INFX q = 0.039; VDZ q = 0.012) and miR-21-5p (pooled q = 0.035; INFX q = 0.027) (Figure 1).

Both reductions persisted at the post-induction time point. miR-376c-3p showed a non-significant trend toward reduction in CD (pooled q = 0.055), while miR-106a-5p, miR-362-3p, and miR-424-5p did not differ from HC at the cohort level.

### 3.3 Treatment-induced change and between-arm comparison

No miRNA showed a paired v1 → v4 change that survived FDR correction within either drug arm, and no miRNA differed between INFX and VDZ at either time point after correction. The complete set of paired within-arm and pooled contrasts (analysis family F2) is reported in Supplementary Table S4.

### 3.4 Concurrent inflammation: post-induction miR-424-5p as a state biomarker

Post-induction miR-424-5p showed the strongest concurrent association with biochemical inflammation in the entire dataset. In the pooled cohort, post-induction miR-424-5p positively correlated with concurrent faecal calprotectin (Spearman *ρ* = +0.52, q = 0.004) and CRP (ρ = +0.44, q = 0.030) (Supplementary Figure S1).

The associations were stronger within the INFX subgroup (FC ρ = +0.62, CRP ρ = +0.63, both q ≈ 0.02) and remained directionally consistent in VDZ; the full set of FDR-significant subgroup-specific associations across the pooled, INFX-only and VDZ-only analyses is provided in Supplementary Figure S2.

A heatmap visualisation of the Spearman correlations between all six panel miRNAs and concurrent or subsequent faecal calprotectin and CRP, across the relevant timepoint pairings, is provided in Supplementary Figure S3, which shows that miR-424-5p stands out as the only miRNA with strong concurrent state-marker correlations while baseline miR-146b-5p and miR-106a-5p emerge as the predictive baseline signals for post-induction CRP. The complete numerical results of this analysis family, across all six miRNAs, three timepoints and all outcomes within the pooled, INFX and VDZ cohorts, are tabulated in Supplementary Table S5. No panel miRNA correlated with baseline SES-CD as a continuous variable (all q = 0.93, *n* = 30; Supplementary Table S6).

Sensitivity analyses preserved the principal miR-424-5p × concurrent inflammation associations: partial Spearman correlations adjusting for each of HBI, SES-CD, CRP and FC at baseline all maintained a strong positive association (smallest adjusted *ρ* = +0.43; largest = +0.57 after baseline-SES-CD adjustment), and leave-one-out single-observation removal as well as alternative single-reference normalisations produced essentially unchanged effect sizes (Supplementary Figure S4). The corresponding numerical values are provided in Supplementary Table S7 for the single-reference normalisations and in Supplementary Table S8 for the leave-one-out analysis. The association also persisted after adjustment for treatment arm (partial rho = +0.53 for faecal calprotectin and +0.50 for CRP) and in a single multivariable model additionally including prior anti-TNF exposure and disease duration (partial rho = +0.52 for faecal calprotectin and +0.51 for CRP); full adjusted values with 95% confidence intervals are provided in Supplementary Table S9.

Post-induction miR-424-5p was not prognostic for 12-month endoscopic activity (pooled Spearman *ρ* = +0.06, *p* = 0.76, *n* = 30), confirming that miR-424-5p in this cohort behaves as a concurrent biochemical state monitor rather than a long-term endoscopic predictor (Supplementary Figure S5).

### 3.5 Predictive associations within the pre-specified analysis family

**Figure 2:**
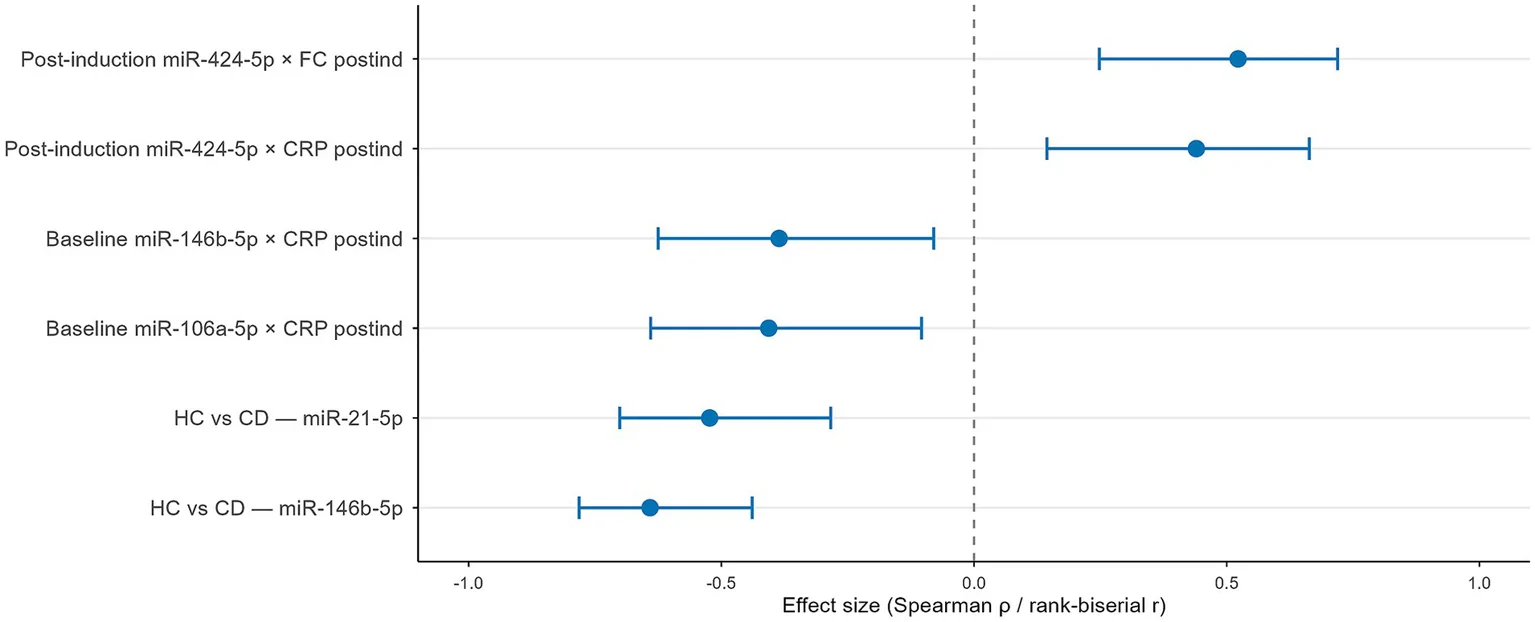
Forest plot consolidating all pooled-cohort FDR-significant findings across the four pre-specified analysis families. The plot includes: HC-vs-CD-pooled contrasts for miR-21-5p (rank-biserial *r* = −0.52, *q* = 0.034, *n* = 49) and miR-146b-5p (*r* = −0.64, *q* = 0.012, *n* = 49); pooled baseline miR-146b-5p × post-induction CRP (Spearman *ρ* = −0.39, *q* = 0.046, *n* = 39); pooled baseline miR-106a-5p × post-induction CRP (*ρ* = −0.41, *q* = 0.046, *n* = 39); pooled post-induction miR-424-5p × concurrent CRP (*ρ* = +0.44, *q* = 0.030, *n* = 39); and pooled post-induction miR-424-5p × concurrent FC (*ρ* = +0.52, *q* = 0.004, *n* = 39). Error bars are 95% confidence intervals from the Fisher z-transform. Dashed line marks zero effect.

In the pooled cohort, baseline miR-146b-5p and miR-106a-5p both inversely correlated with post-induction CRP (ρ ≈ −0.40, q = 0.046 each) (Figure 2; the complete predictive scan is provided in Supplementary Table S5 and the family-wide correction across the entire pooled predictive scan in Supplementary Table S3).

To visualise whether baseline serum miRNA levels differ between eventual responders and non-responders, we stratified the pooled cohort (INFX + VDZ) by two clinically meaningful outcomes and plotted baseline miRNA values in each group (Supplementary Figure S6).

At post-induction, responders were defined by biochemical remission (faecal calprotectin < 250 μg/g; *n* = 29 responders, *n* = 10 non-responders); at 12 months, responders were defined by endoscopic remission (SES-CD < 3; *n* = 18 responders, *n* = 12 non-responders). For the post-induction biochemical outcome, baseline miRNA values were essentially overlapping between groups, with no contrast reaching nominal significance (smallest *p* = 0.085 for miR-424-5p). For the 12-month endoscopic outcome, two miRNAs showed nominally lower baseline levels in patients who achieved endoscopic remission: miR-106a-5p (Mann–Whitney *p* = 0.048) and miR-424-5p (*p* = 0.043). Neither contrast survives FDR correction across the 24 explored comparisons in this analysis (q ≈ 0.5 for both), and these signals are presented as hypothesis-generating rather than as confirmed baseline predictors.

In an exploratory drug-arm subgroup analysis, post-induction miR-146b-5p showed nominal discrimination of treatment success specifically within the VDZ arm (LOO-CV AUC 0.79, 95% CI 0.53–1.00, *n* = 17); the wide confidence interval precludes clinical interpretation without external validation (Supplementary Figure S7). This subgroup analysis is exploratory only; given the small sample it is not interpreted as evidence that miR-146b-5p outperforms faecal calprotectin. Treatment success was also re-analysed under a continuation-only definition, with an essentially identical between-arm pattern (Supplementary Table S10).

### 3.6 Cross-sectional disease activity discrimination

Across 78 pooled patient-timepoints (44 active by HBI, 34 inactive), faecal calprotectin alone achieved leave-one-patient-out cross-validated AUC 0.80 (patient-clustered 95% CI 0.71 to 0.89) for HBI-defined activity (Supplementary Figure S8).

### 3.7 Exploratory phenotype-stratified analysis

The following analysis is explicitly post-hoc and was not part of the four pre-specified analysis families. Patients with Montreal B3 (penetrating) disease (*n* = 19) showed an upregulation of miR-106a-5p over the induction period (median *Δ* v4 − v1 = +0.29 log₂ fold-change), whereas patients with non-penetrating disease (B1 + B2, n = 20) showed no net change (median Δ = −0.06; Mann–Whitney *p* = 0.013, Supplementary Figure S9). To address the post-hoc nature of the cut, we performed a permutation test by shuffling the B3/non-B3 labels 50,000 times and recomputing the mean difference: the two-sided permutation-based *p*-value was 0.019. After adjustment for treatment success via partial Spearman correlation, the association persisted (partial r = +0.37; permutation *p* = 0.042), indicating that the phenotype effect is not entirely a confounded outcome signal in this exploratory analysis. The full phenotype scan across all six miRNAs and three timepoints (18 contrasts with FDR correction), of which this is the only nominally significant contrast, is reported in Supplementary Table S11.

## 4 Discussion

In this longitudinal cohort of patients with active Crohn’s disease undergoing biologic induction with infliximab or vedolizumab, paired pre- and post-induction serum sampling identified a circulating miRNA signature with both predictive and concurrent biomarker properties. To our knowledge, this is the first prospective study to characterise serum miRNA dynamics across a defined induction window in two contrasting biologic mechanisms — TNF inhibition and α4β7 integrin antagonism — within a single pre-specified analytic framework. Four findings constitute the principal contribution.

First, miR-21-5p and miR-146b-5p were significantly reduced in CD relative to healthy controls (q < 0.05), reproducing prior serum-based reports (15) and contrasting with the well-documented elevation of these miRNAs in colonic tissue (14). The directional discordance between blood and tissue is biologically coherent: tissue miRNA elevation reflects local upregulation in inflamed mucosa, whereas circulating levels integrate haematopoietic, endothelial, and tissue-derived pools. The reduction in serum miR-21-5p (PDCD4 and TLR4-pathway negative feedback) and miR-146b-5p (NF-κB negative feedback via IRAK1 and TRAF6) is therefore interpretable as a deficit of anti-inflammatory regulatory capacity in active CD (16). The convergence between our cohort and independent serum-based studies strengthens the case for these miRNAs as components of a reproducible IBD-related serum signature.

Second, post-induction miR-424-5p emerged as the strongest concurrent biomarker of biochemical inflammation in this cohort. The associations with concurrent faecal calprotectin (Spearman *ρ* = +0.52, q = 0.004) and CRP (ρ = +0.44, q = 0.030) were robust across drug arms, persisted under each of four severity adjustments (smallest adjusted ρ = +0.43; largest = +0.57), held under leave-one-out sensitivity, and remained essentially identical under single-reference normalisation. miR-424-5p is an established regulator of profibrotic and macrophage-differentiation programmes across non-IBD organ systems — lung, liver, and oral submucosa (17–19) — through CUL2/HIF-1α–mediated hypoxia signalling and PU.1-driven monocyte commitment. Our findings raise the hypothesis that this mechanistic literature may extend into the circulation in CD and are consistent with miR-424-5p reflecting systemic inflammatory biology; we note, however, that the supporting profibrotic literature derives from non-intestinal organs (lung, liver, oral submucosa) and that we measured neither fibrosis by imaging nor histology, so the transmural-fibrotic interpretation remains a mechanistic hypothesis not verified in our dataset in a manner distinct from the mucosal-luminal signal captured by faecal calprotectin (20). The candidate clinical utility, if validated, lies in this biological distinctiveness: a complementary biochemical readout that may capture aspects of disease activity beyond mucosal inflammation. We stress, however, that because the miR-424-5p signal is largely parallel to faecal calprotectin — which is cheaper and already validated — its present value is best framed as confirmation of biological plausibility rather than as a state biomarker ready for clinical use.

Third, baseline miR-146b-5p and miR-106a-5p inversely predicted post-induction CRP independently of baseline disease severity (partial r = −0.46, *p* = 0.010 for miR-146b-5p; partial r = −0.42, *p* = 0.020 for miR-106a-5p). To our knowledge, this is the first report of severity-independent serum miRNA predictors of biochemical induction response in CD, addressing a clinical question that faecal calprotectin does not adequately answer at the pre-treatment time point. The mechanistic basis is biologically plausible: miR-146b-5p is a canonical NF-κB-pathway negative-feedback element, and miR-106a-5p directly represses IL-10 mRNA and modulates macrophage SIRPα expression (21–23). Lower pre-treatment levels of these regulatory miRNAs may therefore mark patients with attenuated anti-inflammatory feedback capacity who are less likely to achieve biochemical control during induction. The translational implication is direct: a pre-treatment serum measurement could, in principle, identify patients at higher risk of biochemical non-response before the first infusion.

Fourth, in a single pre-declared post-hoc contrast, treatment-induced upregulation of miR-106a-5p was associated with the Montreal B3 (penetrating) phenotype (permutation *p* = 0.019; partial r = +0.37 after adjustment for treatment success, permutation *p* = 0.042). The biological coherence is notable. miR-106a-5p directly targets IL-10 mRNA and modulates macrophage SIRPα-mediated tolerance, and miR-106a deficiency attenuates inflammation in murine colitis (22, 23). Penetrating CD is characterised by transmural fibrosis, fistulising complications, and frequent therapy-refractoriness (2). Differential treatment-induced engagement of the IL-10/SIRPα axis in penetrating disease — if confirmed in independent cohorts — would mechanistically link biologic-induction biology to the well-documented clinical refractoriness of this phenotype, generating a testable hypothesis with direct clinical relevance. We emphasise that this finding is exploratory and post-hoc; it requires independent prospective validation, ideally with parallel cytokine measurement and imaging-based fibrosis assessment.

Taken together, these findings converge on three biologically distinct regulatory axes: NF-κB feedback (miR-146b-5p, miR-21-5p), IL-10/SIRPα macrophage regulation (miR-106a-5p), and hypoxia–fibrosis programmes (miR-424-5p). This convergence has conceptual implications. The dominant non-invasive biomarker paradigm in CD — CRP, faecal calprotectin — collapses biological complexity onto a single intensity dimension. A multi-axis circulating miRNA signature that simultaneously samples regulatory feedback, macrophage tolerance, and fibrotic biology may capture aspects of CD heterogeneity, particularly the penetrating-fibrostenotic phenotype, that single-analyte markers cannot. The present panel was selected for broader IBD biological rationale and is therefore complementary to, rather than overlapping with, the literature-predicted treatment-class biomarkers (miR-126 and miR-486 for vedolizumab response; miR-23a for anti-TNF response; miR-29 for ustekinumab response) (11, 24). Future panels that integrate both candidate-predictor classes within a multiplexed assay would provide a more comprehensive interrogation of the candidate-predictor space.

Two distinct clinical biomarker questions emerge from our analyses. For the question of current disease activity (“is this patient currently inflamed?”), faecal calprotectin remained the dominant non-invasive marker (leave-one-patient-out AUC 0.80, patient-clustered 95% CI 0.71 to 0.89, n = 78 patient-timepoints), with miRNA panel additions providing parallel rather than incremental performance — consistent with the established discriminative power of FC for active mucosal inflammation (25). For the question of treatment response prediction (“will this patient respond?”), our pre-specified analyses identified baseline miR-146b-5p and miR-106a-5p as candidate severity-independent predictors, addressing a question that FC does not adequately answer at the pre-treatment time point (26, 27). The two biomarker classes therefore appear complementary rather than competitive: FC for current disease state, serum miRNAs for response prediction. Validating this complementarity in an independent cohort could enable a multi-modal biomarker strategy in which FC and serum miRNAs jointly inform clinical decisions at distinct time points in the patient pathway.

The principal strengths of this study are the longitudinal paired pre- and post-induction sampling design, which permits within-patient trajectory analysis and minimises between-subject variance; the inclusion of two contrasting biologic mechanisms within a single protocol, supporting cross-mechanism comparison; comprehensive clinical phenotyping including Montreal classification and 12-month endoscopic follow-up; pre-specified analysis families with false discovery rate (FDR) correction for multiple testing across four independent question types, limiting false positives; and explicit sensitivity analyses for severity confounding, leave-one-out stability, and reference-miRNA dependence — the latter rarely reported in serum miRNA work.

The principal limitations are equally identifiable. The sample size (*n* = 39 across two arms of 20 and 19) limits statistical power for drug-specific claims and formal interaction testing; we have therefore presented primary findings using the pooled cohort and labelled drug-stratified analyses as exploratory. The single-centre design without an independent validation cohort is the central translational limitation; recent systematic reviews have identified absence of independent validation as the principal barrier to clinical use of serum miRNA biomarkers in IBD, and our findings are best understood as candidate signals worth prospective replication rather than as confirmed biomarkers (10, 28). The two drug arms differ at baseline in ways that reflect contemporary clinical practice — longer disease duration, higher prior anti-TNF and azathioprine exposure, higher baseline CRP in vedolizumab patients — constraining direct between-arm causal inference; we have correspondingly avoided interpreting null between-arm comparisons as evidence of equivalence. Because biologic choice was not randomised and followed contemporary clinical positioning (vedolizumab preferentially in more refractory, anti-TNF-exposed patients), confounding by indication pervades every drug-stratified analysis. Any apparent infliximab-versus-vedolizumab difference is therefore more plausibly explained by these baseline cohort differences than by a drug-class effect, and none of our between-arm comparisons should be interpreted as evidence of a mechanism-specific effect. A further limitation concerns the reference group: the healthy controls were only ten subjects, recruited from hospital staff and visitors and not formally matched for age, sex or BMI. Because the first result (reduced miR-21-5p and miR-146b-5p in CD versus HC) rests entirely on this comparison, the observed difference may in part reflect imbalance of the reference group rather than disease-specific biology; a formal age- and BMI-adjusted analysis was not feasible given the small control group, and this contrast should be regarded as preliminary.

Three additional methodological notes are reported here in compressed form. First, the choice of paired endogenous references (miR-93-5p, miR-425-5p; geometric mean) was empirically validated within this cohort (geNorm M-value 0.37, well below the 0.5 threshold for excellent stability; no significant Cq shifts between HC vs. CD or paired v1 → v4; convergent headline-finding effect sizes under single-reference normalisation), avoiding the documented pitfalls of RNU6b (unsuitable in blood) and miR-16 (haemolysis-confounded). An exogenous spike-in (cel-miR-39) sensitivity analysis was not performed because of insufficient remaining sample volume. All serum samples were additionally screened for haemolysis before analysis, since free haemoglobin can confound low-to-moderate serum miRNA levels; samples were checked using the miR-23a-3p/miR-451a ratio together with spectrophotometric absorbance at 414 nm (A₄₁₄ > 0.2 absorbance units taken to indicate haemolysis), and visibly or biochemically haemolysed samples were excluded, providing a defined quality-control standard for the miR-21-5p and miR-106a-5p measurements. Second, the composite outcome “treatment success at last follow-up” pools therapy continuation with therapy stopped because of sustained remission; the components are tabulated in Table 2 and a continuation-only sensitivity analysis confirmed essentially identical results, but a time-to-event analysis with formal censoring would be informative in larger cohorts. Third, 12-month SES-CD scores were available for 77% of patients (90% INFX, 63% VDZ); analyses involving 12-month endoscopic outcomes used complete-case data, and we did not measure tissue miRNA or cytokines in parallel, both of which would strengthen the mechanistic interpretation in future replication studies.

The most important next step is independent external validation. Prospective replication of the three principal pre-specified findings — post-induction miR-424-5p × concurrent inflammation, baseline miR-146b-5p × post-induction CRP, and baseline miR-106a-5p × post-induction CRP — in an independent multicentre cohort with pre-registered analysis protocols is the prerequisite for any subsequent clinical translation. Beyond validation, three concrete directions follow naturally from our findings. Mechanistic studies of miR-106a-5p modulation of IL-10 and SIRPα in CD-derived macrophages, particularly in penetrating-phenotype patients with parallel cytokine measurement and cross-sectional imaging-based fibrosis assessment, would test the biological hypothesis arising from the post-hoc B3 finding. Integrative profiling of circulating, tissue, and faecal miRNA within the same patient cohort would clarify the biological provenance of the serum signals we report. And multiplexed panel development that combines our predictive markers with literature-predicted treatment-class biomarkers (miR-126, miR-486, miR-23a, miR-29) within a single multiplexed assay would enable adequately powered investigation of therapy-class–specific prediction.

## 5 Conclusion

In this longitudinal cohort of 39 Crohn’s disease patients receiving biologic induction therapy, we identified two principal serum miRNA biomarker signals: post-induction miR-424-5p as a robust concurrent biomarker of biochemical inflammation, and baseline miR-146b-5p and miR-106a-5p as candidate severity-independent predictors of post-induction CRP. Both associations survived adjustment for baseline disease severity, leave-one-out sensitivity and single-reference normalisation. miR-21-5p and miR-146b-5p were reduced in CD versus healthy controls, replicating prior reports. An exploratory post-hoc analysis linked treatment-induced miR-106a-5p upregulation to the Montreal B3 (penetrating) phenotype, biologically consistent with the established role of this miRNA in IL-10 and SIRPα regulation. These miRNA signals are complementary to faecal calprotectin, which remains the dominant non-invasive marker of cross-sectional disease activity. Prospective independent validation in larger multicentre cohorts is the essential next step before clinical translation.

## Data Availability

The individual-level data underlying this study (Cq and ΔΔCt values and the associated clinical variables) are not publicly available because they constitute potentially identifiable patient data and their sharing was not covered by the original ethics approval and patient consent. De-identified data and the analysis code are available from the corresponding author on reasonable request, subject to a data-sharing agreement and the constraints of the approving ethics committee.
