# Supplementary material for: Circulating microRNAs as biomarkers of disease activity and treatment response in Crohn’s disease patients receiving infliximab or vedolizumab: a longitudinal cohort study
Source: Front Med (Lausanne). 2026 Aug 13;13:1923232. doi: 10.3389/fmed.2026.1923232 (PMC13518191; doi:10.3389/fmed.2026.1923232)
Supplement: Supplementary file 1 [file Table_1.docx]

**Supplementary Tables**

*Circulating microRNAs as biomarkers of disease activity and treatment response in Crohn’s disease patients receiving infliximab or vedolizumab: a longitudinal cohort study*

Manuscript 1923232. Supplementary Tables S1 to S11.

**Supplementary Table S1.** Empirical stability metrics for the paired endogenous reference miRNAs (miR-93-5p and miR-425-5p) used for ΔΔCt normalisation. The geNorm M-value was 0.371, below the 0.5 threshold for excellent stability; neither reference Cq differed significantly between healthy controls and CD patients, nor between paired pre-induction and post-induction samples within patients. Cited in Section 2.2.

| **Metric** | **Value** | **Threshold_or_note** |
| --- | --- | --- |
| geNorm M-value (paired references) | 0.371 | Excellent <0.5; acceptable <1.5 |
| miR-93-5p Cq HC vs CD (Mann-Whitney p) | 0.3 | p > 0.05 = no shift between groups |
| miR-425-5p Cq HC vs CD (Mann-Whitney p) | 0.59 | p > 0.05 = no shift between groups |
| miR-93-5p paired pre- vs post-induction (Wilcoxon p) | 0.77 | p > 0.05 = no shift across induction |
| miR-425-5p paired pre- vs post-induction (Wilcoxon p) | 0.78 | p > 0.05 = no shift across induction |

**Supplementary Table S2.** Rationale for selection of each of the six target miRNAs, mapping each miRNA to (a) the principal prior IBD evidence, (b) the regulatory pathway it engages, and (c) the cellular and tissue context in which dysregulation has been reported. Cited in Section 2.2.

| **miRNA** | **Pathway / cellular role** | **Reported in IBD** | **Tissue / blood compartment** | **Selection rationale** |
| --- | --- | --- | --- | --- |
| miR-21-5p | NF-κB feedback (PDCD4, TLR4); T-cell function; intestinal barrier | Schönauen et al., 2018 (CD serum reduction); Mohammadnia-Afrouzi et al. (CD serum reduction); Wu et al., 2010 (CD colon tissue elevation) | Serum: reduced in active CD. Tissue: elevated in inflamed colon. | Most replicated CD-associated miRNA across studies; compartment-specific direction makes serum-based study informative. |
| miR-146b-5p | NF-κB feedback (IRAK1, TRAF6, RELA targets); innate-immune regulation | Chen et al. (CD activity correlation); reviews in Yarani 2022, Sokal-Dembowska 2025 | Serum and PBMC. | Top NF-κB feedback miRNA in IBD; correlated with CRP and disease activity indices. |
| miR-106a-5p | Translational suppression of IL-10 (Sharma 2009); SIRPα modulation on macrophages (Sanctuary 2019); miR-17-92/106a cluster | Murine IBD: miR-106a deficiency attenuates colitis; CD blood: 3 of 4 prior studies report elevation | Serum and PBMC. | Anti-inflammatory feedback regulator with strong mechanistic link to IL-10 axis; relevant to penetrating CD biology. |
| miR-362-3p | Treg differentiation; chemokine signalling | IBD-blood profiling studies (reviewed in Yarani 2022) | Serum and whole blood. | Treg-axis miRNA included to test for adaptive-immune signatures complementary to NF-κB markers. |
| miR-376c-3p | Wnt-pathway and fibrosis-associated regulation; macrophage activation | Caparrós et al. (CD blood elevation); profibrotic axis literature | Serum and tissue. | Member of profibrotic miRNA cluster; complementary to miR-424-5p for fibrosis-relevant signal in serum. |
| miR-424-5p | Monocyte/myofibroblast differentiation (PU.1 programme); NFI-A inhibition; HIF-1α axis | Wang et al. 2021 (liver fibrosis); Tu et al. 2020 (lung fibrosis); Chou et al. 2023 (oral submucous fibrosis) | Tissue (multiple organs); serum association with fibrosis recently reported. | Strong cross-organ profibrotic effector; tested as candidate transmural-fibrosis biomarker complementing mucosal FC. |

**Supplementary Table S3.** Family-wide Benjamini–Hochberg correction applied across the entire pooled predictive scan (84 tests: six panel miRNAs × two predictor timepoints, baseline and post-induction log2 fold-change, × seven outcomes), reporting Spearman rho, nominal p and family-wide q (q_family). Only the post-induction miR-424-5p × concurrent faecal calprotectin association approached the significance threshold (q = 0.054); the corresponding association with CRP (q = 0.213) and the baseline miR-146b-5p (q = 0.322) and miR-106a-5p (q = 0.288) associations with post-induction CRP did not. Cited in Sections 2.4 and 3.5.

| **miRNA** | **timepoint** | **outcome** | **p** | **rho** | **q_family** |
| --- | --- | --- | --- | --- | --- |
| miR-21-5p | v1_log2FC | FC_postind | 0.788193 | 0.044444 | 0.955554 |
| miR-21-5p | v1_log2FC | CRP_postind | 0.468394 | -0.119581 | 0.945133 |
| miR-21-5p | v1_log2FC | HBI_postind | 0.807274 | 0.04036 | 0.955554 |
| miR-21-5p | v1_log2FC | FC_12m | 0.742599 | 0.055075 | 0.955554 |
| miR-21-5p | v1_log2FC | CRP_12m | 0.301773 | -0.174446 | 0.884159 |
| miR-21-5p | v1_log2FC | SESCD_12m | 0.128016 | -0.284183 | 0.614872 |
| miR-21-5p | v1_log2FC | tx_success | 0.396789 | -0.155055 | 0.945133 |
| miR-21-5p | v4_log2FC | FC_postind | 0.620555 | 0.081802 | 0.955554 |
| miR-21-5p | v4_log2FC | CRP_postind | 0.265645 | 0.182685 | 0.829833 |
| miR-21-5p | v4_log2FC | HBI_postind | 0.710081 | 0.061471 | 0.955554 |
| miR-21-5p | v4_log2FC | FC_12m | 0.741117 | -0.055403 | 0.955554 |
| miR-21-5p | v4_log2FC | CRP_12m | 0.451428 | 0.127672 | 0.945133 |
| miR-21-5p | v4_log2FC | SESCD_12m | 0.406164 | -0.157397 | 0.945133 |
| miR-21-5p | v4_log2FC | tx_success | 0.131758 | -0.272208 | 0.614872 |
| miR-146b-5p | v1_log2FC | FC_postind | 0.676644 | -0.068945 | 0.955554 |
| miR-146b-5p | v1_log2FC | CRP_postind | 0.015321 | -0.385657 | 0.321751 |
| miR-146b-5p | v1_log2FC | HBI_postind | 0.09461 | 0.271444 | 0.529815 |
| miR-146b-5p | v1_log2FC | FC_12m | 0.405806 | -0.138837 | 0.945133 |
| miR-146b-5p | v1_log2FC | CRP_12m | 0.074747 | -0.29651 | 0.510356 |
| miR-146b-5p | v1_log2FC | SESCD_12m | 0.381093 | -0.165849 | 0.945133 |
| miR-146b-5p | v1_log2FC | tx_success | 0.89575 | 0.02412 | 0.977561 |
| miR-146b-5p | v4_log2FC | FC_postind | 0.494233 | 0.112782 | 0.945133 |
| miR-146b-5p | v4_log2FC | CRP_postind | 0.734588 | 0.05607 | 0.955554 |
| miR-146b-5p | v4_log2FC | HBI_postind | 0.305245 | -0.168476 | 0.884159 |
| miR-146b-5p | v4_log2FC | FC_12m | 0.759465 | 0.051352 | 0.955554 |
| miR-146b-5p | v4_log2FC | CRP_12m | 0.146573 | 0.243413 | 0.648007 |
| miR-146b-5p | v4_log2FC | SESCD_12m | 0.941738 | -0.013935 | 0.97852 |
| miR-146b-5p | v4_log2FC | tx_success | 0.164916 | -0.251534 | 0.692645 |
| miR-106a-5p | v1_log2FC | FC_postind | 0.795746 | -0.042825 | 0.955554 |
| miR-106a-5p | v1_log2FC | CRP_postind | 0.010302 | -0.406147 | 0.288468 |
| miR-106a-5p | v1_log2FC | HBI_postind | 0.26408 | -0.183274 | 0.829833 |
| miR-106a-5p | v1_log2FC | FC_12m | 0.428592 | 0.132268 | 0.945133 |
| miR-106a-5p | v1_log2FC | CRP_12m | 0.085059 | -0.286965 | 0.510356 |
| miR-106a-5p | v1_log2FC | SESCD_12m | 0.131571 | 0.28167 | 0.614872 |
| miR-106a-5p | v1_log2FC | tx_success | 0.721848 | 0.065468 | 0.955554 |
| miR-106a-5p | v4_log2FC | FC_postind | 0.027877 | -0.352215 | 0.468326 |
| miR-106a-5p | v4_log2FC | CRP_postind | 0.266732 | -0.182277 | 0.829833 |
| miR-106a-5p | v4_log2FC | HBI_postind | 0.073675 | -0.289658 | 0.510356 |
| miR-106a-5p | v4_log2FC | FC_12m | 0.765943 | 0.049929 | 0.955554 |
| miR-106a-5p | v4_log2FC | CRP_12m | 0.24138 | -0.197475 | 0.829833 |
| miR-106a-5p | v4_log2FC | SESCD_12m | 0.469388 | -0.137294 | 0.945133 |
| miR-106a-5p | v4_log2FC | tx_success | 0.925437 | -0.017228 | 0.97852 |
| miR-362-3p | v1_log2FC | FC_postind | 0.51473 | 0.107517 | 0.945133 |
| miR-362-3p | v1_log2FC | CRP_postind | 0.783908 | 0.045365 | 0.955554 |
| miR-362-3p | v1_log2FC | HBI_postind | 0.518788 | -0.106487 | 0.945133 |
| miR-362-3p | v1_log2FC | FC_12m | 0.976578 | -0.004927 | 0.982042 |
| miR-362-3p | v1_log2FC | CRP_12m | 0.24109 | 0.197594 | 0.829833 |
| miR-362-3p | v1_log2FC | SESCD_12m | 0.772617 | -0.055055 | 0.955554 |
| miR-362-3p | v1_log2FC | tx_success | 0.693908 | -0.072359 | 0.955554 |
| miR-362-3p | v4_log2FC | FC_postind | 0.692572 | 0.067232 | 0.955554 |
| miR-362-3p | v4_log2FC | CRP_postind | 0.889963 | 0.02355 | 0.977561 |
| miR-362-3p | v4_log2FC | HBI_postind | 0.085027 | 0.286993 | 0.510356 |
| miR-362-3p | v4_log2FC | FC_12m | 0.25345 | -0.195389 | 0.829833 |
| miR-362-3p | v4_log2FC | CRP_12m | 0.456197 | 0.130138 | 0.945133 |
| miR-362-3p | v4_log2FC | SESCD_12m | 0.072265 | -0.3449 | 0.510356 |
| miR-362-3p | v4_log2FC | tx_success | 0.514299 | 0.123871 | 0.945133 |
| miR-376c-3p | v1_log2FC | FC_postind | 0.579498 | 0.091521 | 0.955554 |
| miR-376c-3p | v1_log2FC | CRP_postind | 0.369829 | -0.147616 | 0.945133 |
| miR-376c-3p | v1_log2FC | HBI_postind | 0.187958 | 0.215355 | 0.75183 |
| miR-376c-3p | v1_log2FC | FC_12m | 0.040521 | 0.333844 | 0.510356 |
| miR-376c-3p | v1_log2FC | CRP_12m | 0.581353 | -0.093666 | 0.955554 |
| miR-376c-3p | v1_log2FC | SESCD_12m | 0.517619 | -0.122902 | 0.945133 |
| miR-376c-3p | v1_log2FC | tx_success | 0.955222 | 0.010337 | 0.97852 |
| miR-376c-3p | v4_log2FC | FC_postind | 0.856773 | -0.029866 | 0.972553 |
| miR-376c-3p | v4_log2FC | CRP_postind | 0.896098 | -0.021612 | 0.977561 |
| miR-376c-3p | v4_log2FC | HBI_postind | 0.824773 | -0.036634 | 0.962235 |
| miR-376c-3p | v4_log2FC | FC_12m | 0.466895 | 0.121647 | 0.945133 |
| miR-376c-3p | v4_log2FC | CRP_12m | 0.948585 | 0.010977 | 0.97852 |
| miR-376c-3p | v4_log2FC | SESCD_12m | 0.840426 | -0.038378 | 0.967065 |
| miR-376c-3p | v4_log2FC | tx_success | 0.80767 | -0.044794 | 0.955554 |
| miR-424-5p | v1_log2FC | FC_postind | 0.078767 | 0.28489 | 0.510356 |
| miR-424-5p | v1_log2FC | CRP_postind | 0.061266 | 0.30247 | 0.510356 |
| miR-424-5p | v1_log2FC | HBI_postind | 0.743958 | 0.05402 | 0.955554 |
| miR-424-5p | v1_log2FC | FC_12m | 0.713128 | -0.061645 | 0.955554 |
| miR-424-5p | v1_log2FC | CRP_12m | 0.528824 | -0.106911 | 0.945133 |
| miR-424-5p | v1_log2FC | SESCD_12m | 0.082356 | -0.322333 | 0.510356 |
| miR-424-5p | v1_log2FC | tx_success | 0.955222 | 0.010337 | 0.97852 |
| miR-424-5p | v4_log2FC | FC_postind | 0.000644 | 0.522501 | 0.054136 |
| miR-424-5p | v4_log2FC | CRP_postind | 0.005076 | 0.439891 | 0.213184 |
| miR-424-5p | v4_log2FC | HBI_postind | 0.982042 | -0.003726 | 0.982042 |
| miR-424-5p | v4_log2FC | FC_12m | 0.775439 | -0.047848 | 0.955554 |
| miR-424-5p | v4_log2FC | CRP_12m | 0.410702 | 0.139366 | 0.945133 |
| miR-424-5p | v4_log2FC | SESCD_12m | 0.761612 | 0.057796 | 0.955554 |
| miR-424-5p | v4_log2FC | tx_success | 0.778764 | 0.051685 | 0.955554 |

**Supplementary Table S4.** Paired pre-induction (v1) versus post-induction (v4) Wilcoxon signed-rank tests for each of the six panel miRNAs, within each drug arm and in the pooled cohort (analysis family F2), with Benjamini–Hochberg FDR-corrected q-values. No paired contrast survived FDR correction. Cited in Section 3.3.

| **drug** | **mirna** | **n_pairs** | **median_v1** | **median_v4** | **median_change** | **W** | **p** | **q_BH** |
| --- | --- | --- | --- | --- | --- | --- | --- | --- |
| INFX | miR-106a-5p | 20 | -0.046658 | 0.119224 | 0.208865 | 59 | 0.085924 | 0.257772 |
| INFX | miR-146b-5p | 20 | -0.471813 | -0.532178 | 0.001826 | 105 | 1 | 1 |
| INFX | miR-21-5p | 20 | -0.452269 | -0.335719 | 0.102147 | 74 | 0.247145 | 0.370717 |
| INFX | miR-362-3p | 20 | 0.420818 | 0.264756 | -0.021931 | 116 | 0.681322 | 0.817587 |
| INFX | miR-376c-3p | 20 | -1.122276 | -0.983504 | 0.148309 | 74 | 0.247145 | 0.370717 |
| INFX | miR-424-5p | 20 | -0.027383 | -0.35842 | -0.446188 | 159 | 0.043804 | 0.257772 |
| Pooled | miR-106a-5p | 39 | -0.007705 | 0.087476 | 0.130198 | 255 | 0.059575 | 0.216419 |
| Pooled | miR-146b-5p | 39 | -0.581568 | -0.432281 | 0.269705 | 293 | 0.175853 | 0.216419 |
| Pooled | miR-21-5p | 39 | -0.377555 | -0.284155 | 0.110649 | 286 | 0.146691 | 0.216419 |
| Pooled | miR-362-3p | 37 | 0.30738 | 0.217068 | 0.054102 | 364 | 0.850423 | 0.850423 |
| Pooled | miR-376c-3p | 39 | -1.066019 | -0.692246 | 0.189688 | 294 | 0.180349 | 0.216419 |
| Pooled | miR-424-5p | 39 | -0.105629 | -0.397285 | -0.174467 | 510 | 0.094013 | 0.216419 |
| VDZ | miR-106a-5p | 19 | 0.010073 | -0.047325 | 0.116113 | 80 | 0.546088 | 0.819132 |
| VDZ | miR-146b-5p | 19 | -0.735515 | -0.320543 | 0.414291 | 48 | 0.058573 | 0.35144 |
| VDZ | miR-21-5p | 19 | -0.283757 | -0.173107 | 0.110649 | 68 | 0.277241 | 0.554481 |
| VDZ | miR-362-3p | 17 | 0.277778 | 0.185587 | 0.137457 | 75 | 0.943392 | 0.9679 |
| VDZ | miR-376c-3p | 19 | -1.019951 | -0.326159 | 0.415333 | 66 | 0.243201 | 0.554481 |
| VDZ | miR-424-5p | 19 | -0.152823 | -0.45846 | -0.006512 | 96 | 0.9679 | 0.9679 |

**Supplementary Table S5.** Comprehensive results of analysis family F4 (miRNA × outcome predictive associations): each of the six panel miRNAs tested at three predictor timepoints (baseline, post-induction and treatment-induced change Δ = v4 − v1) against each of twelve pre-specified clinical, biochemical and endoscopic outcomes, within the pooled cohort and the INFX and VDZ subgroups (648 tests), with Benjamini–Hochberg FDR correction applied within each cohort × timepoint × outcome cell. The FDR-significant findings reported in the main text are the significant subset of this comprehensive scan. Cited in Sections 3.4 and 3.5.

| **drug** | **predictor** | **mirna** | **outcome** | **test** | **n** | **stat_label** | **stat** | **p** | **q_BH** |
| --- | --- | --- | --- | --- | --- | --- | --- | --- | --- |
| INFX | Baseline | miR-106a-5p | CRP 12m | Spearman | 19 | rho | -0.451 | 0.0526 | 0.3158 |
| INFX | Baseline | miR-146b-5p | CRP 12m | Spearman | 19 | rho | -0.0799 | 0.7451 | 0.8995 |
| INFX | Baseline | miR-21-5p | CRP 12m | Spearman | 19 | rho | 0.0391 | 0.8739 | 0.8995 |
| INFX | Baseline | miR-362-3p | CRP 12m | Spearman | 19 | rho | 0.3489 | 0.1432 | 0.4296 |
| INFX | Baseline | miR-376c-3p | CRP 12m | Spearman | 19 | rho | 0.0311 | 0.8995 | 0.8995 |
| INFX | Baseline | miR-424-5p | CRP 12m | Spearman | 19 | rho | -0.0923 | 0.707 | 0.8995 |
| INFX | Baseline | miR-106a-5p | CRP postind | Spearman | 20 | rho | -0.2548 | 0.2783 | 0.334 |
| INFX | Baseline | miR-146b-5p | CRP postind | Spearman | 20 | rho | -0.3967 | 0.0833 | 0.2198 |
| INFX | Baseline | miR-21-5p | CRP postind | Spearman | 20 | rho | -0.2975 | 0.2027 | 0.304 |
| INFX | Baseline | miR-362-3p | CRP postind | Spearman | 20 | rho | 0 | 1 | 1 |
| INFX | Baseline | miR-376c-3p | CRP postind | Spearman | 20 | rho | -0.3685 | 0.1099 | 0.2198 |
| INFX | Baseline | miR-424-5p | CRP postind | Spearman | 20 | rho | 0.5317 | 0.0158 | 0.095 |
| INFX | Baseline | miR-106a-5p | Clin remission 12m | Wilcoxon | 19 | median(success) - median(fail) | 0.1451 | 0.6171 | 0.6171 |
| INFX | Baseline | miR-146b-5p | Clin remission 12m | Wilcoxon | 19 | median(success) - median(fail) | 0.4017 | 0.1615 | 0.6171 |
| INFX | Baseline | miR-21-5p | Clin remission 12m | Wilcoxon | 19 | median(success) - median(fail) | 0.3113 | 0.3173 | 0.6171 |
| INFX | Baseline | miR-362-3p | Clin remission 12m | Wilcoxon | 19 | median(success) - median(fail) | -0.3006 | 0.3173 | 0.6171 |
| INFX | Baseline | miR-376c-3p | Clin remission 12m | Wilcoxon | 19 | median(success) - median(fail) | 0.4097 | 0.6171 | 0.6171 |
| INFX | Baseline | miR-424-5p | Clin remission 12m | Wilcoxon | 19 | median(success) - median(fail) | -0.1317 | 0.5485 | 0.6171 |
| INFX | Baseline | miR-106a-5p | Clin remission postind | Wilcoxon | 20 | median(success) - median(fail) | 0.0779 | 0.9578 | 0.9578 |
| INFX | Baseline | miR-146b-5p | Clin remission postind | Wilcoxon | 20 | median(success) - median(fail) | -0.0656 | 0.7913 | 0.9496 |
| INFX | Baseline | miR-21-5p | Clin remission postind | Wilcoxon | 20 | median(success) - median(fail) | -0.4649 | 0.153 | 0.459 |
| INFX | Baseline | miR-362-3p | Clin remission postind | Wilcoxon | 20 | median(success) - median(fail) | -0.3639 | 0.4273 | 0.6409 |
| INFX | Baseline | miR-376c-3p | Clin remission postind | Wilcoxon | 20 | median(success) - median(fail) | 1.2222 | 0.4273 | 0.6409 |
| INFX | Baseline | miR-424-5p | Clin remission postind | Wilcoxon | 20 | median(success) - median(fail) | -0.3022 | 0.1248 | 0.459 |
| INFX | Baseline | miR-106a-5p | FC 12m | Spearman | 19 | rho | 0.0912 | 0.7103 | 0.7103 |
| INFX | Baseline | miR-146b-5p | FC 12m | Spearman | 19 | rho | -0.2439 | 0.3144 | 0.5779 |
| INFX | Baseline | miR-21-5p | FC 12m | Spearman | 19 | rho | -0.1772 | 0.468 | 0.5779 |
| INFX | Baseline | miR-362-3p | FC 12m | Spearman | 19 | rho | 0.1737 | 0.477 | 0.5779 |
| INFX | Baseline | miR-376c-3p | FC 12m | Spearman | 19 | rho | 0.3474 | 0.1451 | 0.5779 |
| INFX | Baseline | miR-424-5p | FC 12m | Spearman | 19 | rho | -0.1719 | 0.4815 | 0.5779 |
| INFX | Baseline | miR-106a-5p | FC postind | Spearman | 20 | rho | 0.1332 | 0.5756 | 0.7546 |
| INFX | Baseline | miR-146b-5p | FC postind | Spearman | 20 | rho | -0.1204 | 0.6131 | 0.7546 |
| INFX | Baseline | miR-21-5p | FC postind | Spearman | 20 | rho | -0.1339 | 0.5735 | 0.7546 |
| INFX | Baseline | miR-362-3p | FC postind | Spearman | 20 | rho | -0.009 | 0.9699 | 0.9699 |
| INFX | Baseline | miR-376c-3p | FC postind | Spearman | 20 | rho | 0.1151 | 0.6289 | 0.7546 |
| INFX | Baseline | miR-424-5p | FC postind | Spearman | 20 | rho | 0.3371 | 0.1461 | 0.7546 |
| INFX | Baseline | miR-106a-5p | FC remission 12m | Wilcoxon | 19 | median(success) - median(fail) | 0.2069 | 0.3347 | 0.4016 |
| INFX | Baseline | miR-146b-5p | FC remission 12m | Wilcoxon | 19 | median(success) - median(fail) | 0.3207 | 0.1605 | 0.4016 |
| INFX | Baseline | miR-21-5p | FC remission 12m | Wilcoxon | 19 | median(success) - median(fail) | 0.1996 | 0.4829 | 0.4829 |
| INFX | Baseline | miR-362-3p | FC remission 12m | Wilcoxon | 19 | median(success) - median(fail) | -0.3898 | 0.2542 | 0.4016 |
| INFX | Baseline | miR-376c-3p | FC remission 12m | Wilcoxon | 19 | median(success) - median(fail) | -0.973 | 0.1605 | 0.4016 |
| INFX | Baseline | miR-424-5p | FC remission 12m | Wilcoxon | 19 | median(success) - median(fail) | 0.1906 | 0.2926 | 0.4016 |
| INFX | Baseline | miR-106a-5p | FC remission postind | Wilcoxon | 20 | median(success) - median(fail) | -0.1591 | 0.5637 | 0.8161 |
| INFX | Baseline | miR-146b-5p | FC remission postind | Wilcoxon | 20 | median(success) - median(fail) | 0.1419 | 0.9343 | 0.9343 |
| INFX | Baseline | miR-21-5p | FC remission postind | Wilcoxon | 20 | median(success) - median(fail) | 0.3744 | 0.0833 | 0.4996 |
| INFX | Baseline | miR-362-3p | FC remission postind | Wilcoxon | 20 | median(success) - median(fail) | 0.083 | 0.4095 | 0.8161 |
| INFX | Baseline | miR-376c-3p | FC remission postind | Wilcoxon | 20 | median(success) - median(fail) | -0.3596 | 0.6801 | 0.8161 |
| INFX | Baseline | miR-424-5p | FC remission postind | Wilcoxon | 20 | median(success) - median(fail) | -0.2746 | 0.6207 | 0.8161 |
| INFX | Baseline | miR-106a-5p | HBI 12m | Spearman | 19 | rho | 0.1551 | 0.5261 | 0.7804 |
| INFX | Baseline | miR-146b-5p | HBI 12m | Spearman | 19 | rho | -0.082 | 0.7384 | 0.7804 |
| INFX | Baseline | miR-21-5p | HBI 12m | Spearman | 19 | rho | -0.0685 | 0.7804 | 0.7804 |
| INFX | Baseline | miR-362-3p | HBI 12m | Spearman | 19 | rho | 0.422 | 0.0719 | 0.4316 |
| INFX | Baseline | miR-376c-3p | HBI 12m | Spearman | 19 | rho | -0.0938 | 0.7026 | 0.7804 |
| INFX | Baseline | miR-424-5p | HBI 12m | Spearman | 19 | rho | -0.1073 | 0.662 | 0.7804 |
| INFX | Baseline | miR-106a-5p | HBI postind | Spearman | 20 | rho | -0.0502 | 0.8334 | 0.9819 |
| INFX | Baseline | miR-146b-5p | HBI postind | Spearman | 20 | rho | 0.3215 | 0.1669 | 0.5171 |
| INFX | Baseline | miR-21-5p | HBI postind | Spearman | 20 | rho | 0.3176 | 0.1724 | 0.5171 |
| INFX | Baseline | miR-362-3p | HBI postind | Spearman | 20 | rho | 0.0054 | 0.9819 | 0.9819 |
| INFX | Baseline | miR-376c-3p | HBI postind | Spearman | 20 | rho | 0.2326 | 0.3237 | 0.5606 |
| INFX | Baseline | miR-424-5p | HBI postind | Spearman | 20 | rho | 0.2102 | 0.3737 | 0.5606 |
| INFX | Baseline | miR-106a-5p | SES-CD 12m | Spearman | 18 | rho | 0.3834 | 0.1163 | 0.3262 |
| INFX | Baseline | miR-146b-5p | SES-CD 12m | Spearman | 18 | rho | -0.2913 | 0.2409 | 0.3613 |
| INFX | Baseline | miR-21-5p | SES-CD 12m | Spearman | 18 | rho | -0.4191 | 0.0834 | 0.3262 |
| INFX | Baseline | miR-362-3p | SES-CD 12m | Spearman | 18 | rho | 0.1213 | 0.6316 | 0.6909 |
| INFX | Baseline | miR-376c-3p | SES-CD 12m | Spearman | 18 | rho | -0.1007 | 0.6909 | 0.6909 |
| INFX | Baseline | miR-424-5p | SES-CD 12m | Spearman | 18 | rho | -0.3433 | 0.1631 | 0.3262 |
| INFX | Baseline | miR-106a-5p | Treatment success | Wilcoxon | 15 | median(success) - median(fail) | 0.1688 | 0.5139 | 0.5139 |
| INFX | Baseline | miR-146b-5p | Treatment success | Wilcoxon | 15 | median(success) - median(fail) | 0.5534 | 0.0896 | 0.3019 |
| INFX | Baseline | miR-21-5p | Treatment success | Wilcoxon | 15 | median(success) - median(fail) | 0.279 | 0.5139 | 0.5139 |
| INFX | Baseline | miR-362-3p | Treatment success | Wilcoxon | 15 | median(success) - median(fail) | -0.4766 | 0.1172 | 0.3019 |
| INFX | Baseline | miR-376c-3p | Treatment success | Wilcoxon | 15 | median(success) - median(fail) | 0.9147 | 0.24 | 0.36 |
| INFX | Baseline | miR-424-5p | Treatment success | Wilcoxon | 15 | median(success) - median(fail) | -0.3763 | 0.151 | 0.3019 |
| INFX | Delta | miR-106a-5p | CRP 12m | Spearman | 19 | rho | 0.4483 | 0.0542 | 0.3253 |
| INFX | Delta | miR-146b-5p | CRP 12m | Spearman | 19 | rho | 0.2601 | 0.2822 | 0.8465 |
| INFX | Delta | miR-21-5p | CRP 12m | Spearman | 19 | rho | 0.0133 | 0.9569 | 0.9942 |
| INFX | Delta | miR-362-3p | CRP 12m | Spearman | 19 | rho | 0.0018 | 0.9942 | 0.9942 |
| INFX | Delta | miR-376c-3p | CRP 12m | Spearman | 19 | rho | 0.182 | 0.4559 | 0.9117 |
| INFX | Delta | miR-424-5p | CRP 12m | Spearman | 19 | rho | 0.0266 | 0.9138 | 0.9942 |
| INFX | Delta | miR-106a-5p | CRP postind | Spearman | 20 | rho | 0.1625 | 0.4937 | 0.9108 |
| INFX | Delta | miR-146b-5p | CRP postind | Spearman | 20 | rho | 0.1602 | 0.4999 | 0.9108 |
| INFX | Delta | miR-21-5p | CRP postind | Spearman | 20 | rho | 0.0923 | 0.6987 | 0.9108 |
| INFX | Delta | miR-362-3p | CRP postind | Spearman | 20 | rho | 0 | 1 | 1 |
| INFX | Delta | miR-376c-3p | CRP postind | Spearman | 20 | rho | 0.0732 | 0.759 | 0.9108 |
| INFX | Delta | miR-424-5p | CRP postind | Spearman | 20 | rho | 0.2243 | 0.3418 | 0.9108 |
| INFX | Delta | miR-106a-5p | Clin remission 12m | Wilcoxon | 19 | median(success) - median(fail) | -0.3343 | 0.3173 | 0.476 |
| INFX | Delta | miR-146b-5p | Clin remission 12m | Wilcoxon | 19 | median(success) - median(fail) | 0.5532 | 0.2301 | 0.4603 |
| INFX | Delta | miR-21-5p | Clin remission 12m | Wilcoxon | 19 | median(success) - median(fail) | -0.3631 | 0.1615 | 0.4603 |
| INFX | Delta | miR-362-3p | Clin remission 12m | Wilcoxon | 19 | median(success) - median(fail) | 0.1085 | 0.7642 | 0.7642 |
| INFX | Delta | miR-376c-3p | Clin remission 12m | Wilcoxon | 19 | median(success) - median(fail) | -0.176 | 0.7642 | 0.7642 |
| INFX | Delta | miR-424-5p | Clin remission 12m | Wilcoxon | 19 | median(success) - median(fail) | 0.3889 | 0.0891 | 0.4603 |
| INFX | Delta | miR-106a-5p | Clin remission postind | Wilcoxon | 20 | median(success) - median(fail) | 0.1573 | 0.711 | 0.9496 |
| INFX | Delta | miR-146b-5p | Clin remission postind | Wilcoxon | 20 | median(success) - median(fail) | 0.5164 | 0.2235 | 0.9496 |
| INFX | Delta | miR-21-5p | Clin remission postind | Wilcoxon | 20 | median(success) - median(fail) | 0.1543 | 0.4914 | 0.9496 |
| INFX | Delta | miR-362-3p | Clin remission postind | Wilcoxon | 20 | median(success) - median(fail) | -0.1521 | 0.7913 | 0.9496 |
| INFX | Delta | miR-376c-3p | Clin remission postind | Wilcoxon | 20 | median(success) - median(fail) | 0.1224 | 0.6338 | 0.9496 |
| INFX | Delta | miR-424-5p | Clin remission postind | Wilcoxon | 20 | median(success) - median(fail) | -0.2657 | 0.9578 | 0.9578 |
| INFX | Delta | miR-106a-5p | FC 12m | Spearman | 19 | rho | -0.1053 | 0.668 | 0.7426 |
| INFX | Delta | miR-146b-5p | FC 12m | Spearman | 19 | rho | 0.1333 | 0.5863 | 0.7426 |
| INFX | Delta | miR-21-5p | FC 12m | Spearman | 19 | rho | 0.1018 | 0.6785 | 0.7426 |
| INFX | Delta | miR-362-3p | FC 12m | Spearman | 19 | rho | -0.0807 | 0.7426 | 0.7426 |
| INFX | Delta | miR-376c-3p | FC 12m | Spearman | 19 | rho | -0.0982 | 0.6891 | 0.7426 |
| INFX | Delta | miR-424-5p | FC 12m | Spearman | 19 | rho | 0.1895 | 0.4372 | 0.7426 |
| INFX | Delta | miR-106a-5p | FC postind | Spearman | 20 | rho | -0.222 | 0.3469 | 0.6938 |
| INFX | Delta | miR-146b-5p | FC postind | Spearman | 20 | rho | 0.0625 | 0.7937 | 0.9222 |
| INFX | Delta | miR-21-5p | FC postind | Spearman | 20 | rho | -0.1445 | 0.5434 | 0.8151 |
| INFX | Delta | miR-362-3p | FC postind | Spearman | 20 | rho | -0.0233 | 0.9222 | 0.9222 |
| INFX | Delta | miR-376c-3p | FC postind | Spearman | 20 | rho | -0.3973 | 0.0828 | 0.2485 |
| INFX | Delta | miR-424-5p | FC postind | Spearman | 20 | rho | 0.4251 | 0.0617 | 0.2485 |
| INFX | Delta | miR-106a-5p | FC remission 12m | Wilcoxon | 19 | median(success) - median(fail) | -0.0977 | 0.661 | 0.8608 |
| INFX | Delta | miR-146b-5p | FC remission 12m | Wilcoxon | 19 | median(success) - median(fail) | -0.4593 | 0.7257 | 0.8608 |
| INFX | Delta | miR-21-5p | FC remission 12m | Wilcoxon | 19 | median(success) - median(fail) | 0.1292 | 0.7257 | 0.8608 |
| INFX | Delta | miR-362-3p | FC remission 12m | Wilcoxon | 19 | median(success) - median(fail) | 0.0783 | 0.7925 | 0.8608 |
| INFX | Delta | miR-376c-3p | FC remission 12m | Wilcoxon | 19 | median(success) - median(fail) | -0.2725 | 0.8608 | 0.8608 |
| INFX | Delta | miR-424-5p | FC remission 12m | Wilcoxon | 19 | median(success) - median(fail) | -0.378 | 0.4299 | 0.8608 |
| INFX | Delta | miR-106a-5p | FC remission postind | Wilcoxon | 20 | median(success) - median(fail) | 0.4022 | 0.1376 | 0.4129 |
| INFX | Delta | miR-146b-5p | FC remission postind | Wilcoxon | 20 | median(success) - median(fail) | 0.2368 | 0.7415 | 0.7415 |
| INFX | Delta | miR-21-5p | FC remission postind | Wilcoxon | 20 | median(success) - median(fail) | -0.022 | 0.7415 | 0.7415 |
| INFX | Delta | miR-362-3p | FC remission postind | Wilcoxon | 20 | median(success) - median(fail) | -0.2005 | 0.5094 | 0.7415 |
| INFX | Delta | miR-376c-3p | FC remission postind | Wilcoxon | 20 | median(success) - median(fail) | 0.5094 | 0.216 | 0.432 |
| INFX | Delta | miR-424-5p | FC remission postind | Wilcoxon | 20 | median(success) - median(fail) | -0.6955 | 0.0392 | 0.2353 |
| INFX | Delta | miR-106a-5p | HBI 12m | Spearman | 19 | rho | 0.0063 | 0.9795 | 0.9795 |
| INFX | Delta | miR-146b-5p | HBI 12m | Spearman | 19 | rho | -0.3507 | 0.1409 | 0.8456 |
| INFX | Delta | miR-21-5p | HBI 12m | Spearman | 19 | rho | 0.2119 | 0.3838 | 0.953 |
| INFX | Delta | miR-362-3p | HBI 12m | Spearman | 19 | rho | -0.1722 | 0.4808 | 0.953 |
| INFX | Delta | miR-376c-3p | HBI 12m | Spearman | 19 | rho | -0.1163 | 0.6354 | 0.953 |
| INFX | Delta | miR-424-5p | HBI 12m | Spearman | 19 | rho | -0.055 | 0.823 | 0.9795 |
| INFX | Delta | miR-106a-5p | HBI postind | Spearman | 20 | rho | -0.0487 | 0.8385 | 0.8385 |
| INFX | Delta | miR-146b-5p | HBI postind | Spearman | 20 | rho | -0.4699 | 0.0366 | 0.1599 |
| INFX | Delta | miR-21-5p | HBI postind | Spearman | 20 | rho | -0.2751 | 0.2404 | 0.4808 |
| INFX | Delta | miR-362-3p | HBI postind | Spearman | 20 | rho | 0.2071 | 0.381 | 0.5714 |
| INFX | Delta | miR-376c-3p | HBI postind | Spearman | 20 | rho | -0.4382 | 0.0533 | 0.1599 |
| INFX | Delta | miR-424-5p | HBI postind | Spearman | 20 | rho | -0.1167 | 0.6242 | 0.749 |
| INFX | Delta | miR-106a-5p | SES-CD 12m | Spearman | 18 | rho | -0.3953 | 0.1045 | 0.3135 |
| INFX | Delta | miR-146b-5p | SES-CD 12m | Spearman | 18 | rho | 0.222 | 0.376 | 0.4357 |
| INFX | Delta | miR-21-5p | SES-CD 12m | Spearman | 18 | rho | 0.3281 | 0.1837 | 0.3675 |
| INFX | Delta | miR-362-3p | SES-CD 12m | Spearman | 18 | rho | -0.196 | 0.4357 | 0.4357 |
| INFX | Delta | miR-376c-3p | SES-CD 12m | Spearman | 18 | rho | 0.2285 | 0.3618 | 0.4357 |
| INFX | Delta | miR-424-5p | SES-CD 12m | Spearman | 18 | rho | 0.4667 | 0.0509 | 0.3052 |
| INFX | Delta | miR-106a-5p | Treatment success | Wilcoxon | 15 | median(success) - median(fail) | -0.2387 | 0.2963 | 0.7215 |
| INFX | Delta | miR-146b-5p | Treatment success | Wilcoxon | 15 | median(success) - median(fail) | -0.0432 | 0.794 | 0.794 |
| INFX | Delta | miR-21-5p | Treatment success | Wilcoxon | 15 | median(success) - median(fail) | -0.275 | 0.3608 | 0.7215 |
| INFX | Delta | miR-362-3p | Treatment success | Wilcoxon | 15 | median(success) - median(fail) | 0.4118 | 0.0896 | 0.5379 |
| INFX | Delta | miR-376c-3p | Treatment success | Wilcoxon | 15 | median(success) - median(fail) | -0.3559 | 0.6015 | 0.7218 |
| INFX | Delta | miR-424-5p | Treatment success | Wilcoxon | 15 | median(success) - median(fail) | 0.0811 | 0.5139 | 0.7218 |
| INFX | Post-induction | miR-106a-5p | CRP 12m | Spearman | 19 | rho | 0.214 | 0.3791 | 0.7582 |
| INFX | Post-induction | miR-146b-5p | CRP 12m | Spearman | 19 | rho | 0.214 | 0.3791 | 0.7582 |
| INFX | Post-induction | miR-21-5p | CRP 12m | Spearman | 19 | rho | 0.0923 | 0.707 | 0.9856 |
| INFX | Post-induction | miR-362-3p | CRP 12m | Spearman | 19 | rho | 0.2646 | 0.2737 | 0.7582 |
| INFX | Post-induction | miR-376c-3p | CRP 12m | Spearman | 19 | rho | -0.0044 | 0.9856 | 0.9856 |
| INFX | Post-induction | miR-424-5p | CRP 12m | Spearman | 19 | rho | 0.032 | 0.8967 | 0.9856 |
| INFX | Post-induction | miR-106a-5p | CRP postind | Spearman | 20 | rho | -0.0694 | 0.7712 | 0.7712 |
| INFX | Post-induction | miR-146b-5p | CRP postind | Spearman | 20 | rho | -0.1175 | 0.6218 | 0.7712 |
| INFX | Post-induction | miR-21-5p | CRP postind | Spearman | 20 | rho | -0.0923 | 0.6987 | 0.7712 |
| INFX | Post-induction | miR-362-3p | CRP postind | Spearman | 20 | rho | 0.0809 | 0.7347 | 0.7712 |
| INFX | Post-induction | miR-376c-3p | CRP postind | Spearman | 20 | rho | -0.2777 | 0.2359 | 0.7076 |
| INFX | Post-induction | miR-424-5p | CRP postind | Spearman | 20 | rho | 0.6324 | 0.0028 | 0.0166 |
| INFX | Post-induction | miR-106a-5p | Clin remission 12m | Wilcoxon | 19 | median(success) - median(fail) | -0.2014 | 0.2301 | 0.6356 |
| INFX | Post-induction | miR-146b-5p | Clin remission 12m | Wilcoxon | 19 | median(success) - median(fail) | 0.8244 | 0.0574 | 0.3446 |
| INFX | Post-induction | miR-21-5p | Clin remission 12m | Wilcoxon | 19 | median(success) - median(fail) | -0.1174 | 0.7642 | 0.7642 |
| INFX | Post-induction | miR-362-3p | Clin remission 12m | Wilcoxon | 19 | median(success) - median(fail) | -0.2057 | 0.6171 | 0.7405 |
| INFX | Post-induction | miR-376c-3p | Clin remission 12m | Wilcoxon | 19 | median(success) - median(fail) | 0.4522 | 0.4237 | 0.6356 |
| INFX | Post-induction | miR-424-5p | Clin remission 12m | Wilcoxon | 19 | median(success) - median(fail) | 0.1832 | 0.3681 | 0.6356 |
| INFX | Post-induction | miR-106a-5p | Clin remission postind | Wilcoxon | 20 | median(success) - median(fail) | 0.1057 | 0.2664 | 0.4419 |
| INFX | Post-induction | miR-146b-5p | Clin remission postind | Wilcoxon | 20 | median(success) - median(fail) | 0.2201 | 0.7913 | 0.7913 |
| INFX | Post-induction | miR-21-5p | Clin remission postind | Wilcoxon | 20 | median(success) - median(fail) | -0.1586 | 0.3683 | 0.4419 |
| INFX | Post-induction | miR-362-3p | Clin remission postind | Wilcoxon | 20 | median(success) - median(fail) | -0.5967 | 0.1248 | 0.3745 |
| INFX | Post-induction | miR-376c-3p | Clin remission postind | Wilcoxon | 20 | median(success) - median(fail) | 0.8687 | 0.0807 | 0.3745 |
| INFX | Post-induction | miR-424-5p | Clin remission postind | Wilcoxon | 20 | median(success) - median(fail) | -0.3034 | 0.3683 | 0.4419 |
| INFX | Post-induction | miR-106a-5p | FC 12m | Spearman | 19 | rho | 0.0053 | 0.9829 | 0.9829 |
| INFX | Post-induction | miR-146b-5p | FC 12m | Spearman | 19 | rho | -0.0912 | 0.7103 | 0.9829 |
| INFX | Post-induction | miR-21-5p | FC 12m | Spearman | 19 | rho | -0.0246 | 0.9205 | 0.9829 |
| INFX | Post-induction | miR-362-3p | FC 12m | Spearman | 19 | rho | -0.0175 | 0.9432 | 0.9829 |
| INFX | Post-induction | miR-376c-3p | FC 12m | Spearman | 19 | rho | 0.207 | 0.3951 | 0.9829 |
| INFX | Post-induction | miR-424-5p | FC 12m | Spearman | 19 | rho | 0.2404 | 0.3216 | 0.9829 |
| INFX | Post-induction | miR-106a-5p | FC postind | Spearman | 20 | rho | -0.3837 | 0.0949 | 0.2846 |
| INFX | Post-induction | miR-146b-5p | FC postind | Spearman | 20 | rho | 0.0173 | 0.9423 | 0.9423 |
| INFX | Post-induction | miR-21-5p | FC postind | Spearman | 20 | rho | -0.1716 | 0.4695 | 0.8279 |
| INFX | Post-induction | miR-362-3p | FC postind | Spearman | 20 | rho | 0.0617 | 0.7961 | 0.9423 |
| INFX | Post-induction | miR-376c-3p | FC postind | Spearman | 20 | rho | -0.1415 | 0.5519 | 0.8279 |
| INFX | Post-induction | miR-424-5p | FC postind | Spearman | 20 | rho | 0.623 | 0.0033 | 0.02 |
| INFX | Post-induction | miR-106a-5p | FC remission 12m | Wilcoxon | 19 | median(success) - median(fail) | 0.0842 | 0.7925 | 0.8608 |
| INFX | Post-induction | miR-146b-5p | FC remission 12m | Wilcoxon | 19 | median(success) - median(fail) | 0.468 | 0.4299 | 0.8089 |
| INFX | Post-induction | miR-21-5p | FC remission 12m | Wilcoxon | 19 | median(success) - median(fail) | -0.1071 | 0.8608 | 0.8608 |
| INFX | Post-induction | miR-362-3p | FC remission 12m | Wilcoxon | 19 | median(success) - median(fail) | -0.3588 | 0.2542 | 0.8089 |
| INFX | Post-induction | miR-376c-3p | FC remission 12m | Wilcoxon | 19 | median(success) - median(fail) | -0.3696 | 0.5393 | 0.8089 |
| INFX | Post-induction | miR-424-5p | FC remission 12m | Wilcoxon | 19 | median(success) - median(fail) | -0.2909 | 0.4829 | 0.8089 |
| INFX | Post-induction | miR-106a-5p | FC remission postind | Wilcoxon | 20 | median(success) - median(fail) | 0.3041 | 0.0209 | 0.1255 |
| INFX | Post-induction | miR-146b-5p | FC remission postind | Wilcoxon | 20 | median(success) - median(fail) | 0.2281 | 0.7415 | 0.869 |
| INFX | Post-induction | miR-21-5p | FC remission postind | Wilcoxon | 20 | median(success) - median(fail) | 0.2286 | 0.3223 | 0.6446 |
| INFX | Post-induction | miR-362-3p | FC remission postind | Wilcoxon | 20 | median(success) - median(fail) | -0.0228 | 0.869 | 0.869 |
| INFX | Post-induction | miR-376c-3p | FC remission postind | Wilcoxon | 20 | median(success) - median(fail) | 0.1733 | 0.5637 | 0.8456 |
| INFX | Post-induction | miR-424-5p | FC remission postind | Wilcoxon | 20 | median(success) - median(fail) | -0.7187 | 0.0478 | 0.1433 |
| INFX | Post-induction | miR-106a-5p | HBI 12m | Spearman | 19 | rho | 0.1632 | 0.5044 | 0.6639 |
| INFX | Post-induction | miR-146b-5p | HBI 12m | Spearman | 19 | rho | -0.3336 | 0.1628 | 0.6639 |
| INFX | Post-induction | miR-21-5p | HBI 12m | Spearman | 19 | rho | 0.1821 | 0.4555 | 0.6639 |
| INFX | Post-induction | miR-362-3p | HBI 12m | Spearman | 19 | rho | 0.0884 | 0.7191 | 0.7191 |
| INFX | Post-induction | miR-376c-3p | HBI 12m | Spearman | 19 | rho | -0.2615 | 0.2795 | 0.6639 |
| INFX | Post-induction | miR-424-5p | HBI 12m | Spearman | 19 | rho | -0.1452 | 0.5532 | 0.6639 |
| INFX | Post-induction | miR-106a-5p | HBI postind | Spearman | 20 | rho | -0.3516 | 0.1284 | 0.638 |
| INFX | Post-induction | miR-146b-5p | HBI postind | Spearman | 20 | rho | -0.0951 | 0.6902 | 0.7857 |
| INFX | Post-induction | miR-21-5p | HBI postind | Spearman | 20 | rho | 0.0649 | 0.7857 | 0.7857 |
| INFX | Post-induction | miR-362-3p | HBI postind | Spearman | 20 | rho | 0.2913 | 0.2127 | 0.638 |
| INFX | Post-induction | miR-376c-3p | HBI postind | Spearman | 20 | rho | -0.2002 | 0.3975 | 0.7857 |
| INFX | Post-induction | miR-424-5p | HBI postind | Spearman | 20 | rho | -0.1314 | 0.5809 | 0.7857 |
| INFX | Post-induction | miR-106a-5p | SES-CD 12m | Spearman | 18 | rho | -0.2339 | 0.3502 | 0.7712 |
| INFX | Post-induction | miR-146b-5p | SES-CD 12m | Spearman | 18 | rho | -0.1332 | 0.5983 | 0.8974 |
| INFX | Post-induction | miR-21-5p | SES-CD 12m | Spearman | 18 | rho | -0.0379 | 0.8813 | 0.9422 |
| INFX | Post-induction | miR-362-3p | SES-CD 12m | Spearman | 18 | rho | -0.2177 | 0.3856 | 0.7712 |
| INFX | Post-induction | miR-376c-3p | SES-CD 12m | Spearman | 18 | rho | 0.0184 | 0.9422 | 0.9422 |
| INFX | Post-induction | miR-424-5p | SES-CD 12m | Spearman | 18 | rho | 0.3682 | 0.1327 | 0.7712 |
| INFX | Post-induction | miR-106a-5p | Treatment success | Wilcoxon | 15 | median(success) - median(fail) | -0.2166 | 0.3608 | 0.6015 |
| INFX | Post-induction | miR-146b-5p | Treatment success | Wilcoxon | 15 | median(success) - median(fail) | 0.5378 | 0.2963 | 0.6015 |
| INFX | Post-induction | miR-21-5p | Treatment success | Wilcoxon | 15 | median(success) - median(fail) | -0.2932 | 0.2963 | 0.6015 |
| INFX | Post-induction | miR-362-3p | Treatment success | Wilcoxon | 15 | median(success) - median(fail) | 0.1956 | 0.5139 | 0.6015 |
| INFX | Post-induction | miR-376c-3p | Treatment success | Wilcoxon | 15 | median(success) - median(fail) | 0.5109 | 0.6015 | 0.6015 |
| INFX | Post-induction | miR-424-5p | Treatment success | Wilcoxon | 15 | median(success) - median(fail) | -0.2979 | 0.5139 | 0.6015 |
| Pooled | Baseline | miR-106a-5p | CRP 12m | Spearman | 37 | rho | -0.287 | 0.0851 | 0.2552 |
| Pooled | Baseline | miR-146b-5p | CRP 12m | Spearman | 37 | rho | -0.2965 | 0.0747 | 0.2552 |
| Pooled | Baseline | miR-21-5p | CRP 12m | Spearman | 37 | rho | -0.1744 | 0.3018 | 0.4527 |
| Pooled | Baseline | miR-362-3p | CRP 12m | Spearman | 37 | rho | 0.1976 | 0.2411 | 0.4527 |
| Pooled | Baseline | miR-376c-3p | CRP 12m | Spearman | 37 | rho | -0.0937 | 0.5814 | 0.5814 |
| Pooled | Baseline | miR-424-5p | CRP 12m | Spearman | 37 | rho | -0.1069 | 0.5288 | 0.5814 |
| Pooled | Baseline | miR-106a-5p | CRP postind | Spearman | 39 | rho | -0.4061 | 0.0103 | 0.046 |
| Pooled | Baseline | miR-146b-5p | CRP postind | Spearman | 39 | rho | -0.3857 | 0.0153 | 0.046 |
| Pooled | Baseline | miR-21-5p | CRP postind | Spearman | 39 | rho | -0.1196 | 0.4684 | 0.5621 |
| Pooled | Baseline | miR-362-3p | CRP postind | Spearman | 39 | rho | 0.0454 | 0.7839 | 0.7839 |
| Pooled | Baseline | miR-376c-3p | CRP postind | Spearman | 39 | rho | -0.1476 | 0.3698 | 0.5547 |
| Pooled | Baseline | miR-424-5p | CRP postind | Spearman | 39 | rho | 0.3025 | 0.0613 | 0.1225 |
| Pooled | Baseline | miR-106a-5p | Clin remission 12m | Wilcoxon | 35 | median(success) - median(fail) | 0.1531 | 0.4095 | 0.8456 |
| Pooled | Baseline | miR-146b-5p | Clin remission 12m | Wilcoxon | 35 | median(success) - median(fail) | 0.2564 | 0.5637 | 0.8456 |
| Pooled | Baseline | miR-21-5p | Clin remission 12m | Wilcoxon | 35 | median(success) - median(fail) | 0.1806 | 0.3865 | 0.8456 |
| Pooled | Baseline | miR-362-3p | Clin remission 12m | Wilcoxon | 35 | median(success) - median(fail) | 0.1254 | 0.9015 | 0.9015 |
| Pooled | Baseline | miR-376c-3p | Clin remission 12m | Wilcoxon | 35 | median(success) - median(fail) | 0.0905 | 0.4333 | 0.8456 |
| Pooled | Baseline | miR-424-5p | Clin remission 12m | Wilcoxon | 35 | median(success) - median(fail) | 0.0113 | 0.7415 | 0.8898 |
| Pooled | Baseline | miR-106a-5p | Clin remission postind | Wilcoxon | 39 | median(success) - median(fail) | 0.4027 | 0.0798 | 0.479 |
| Pooled | Baseline | miR-146b-5p | Clin remission postind | Wilcoxon | 39 | median(success) - median(fail) | -0.1659 | 0.7555 | 0.9066 |
| Pooled | Baseline | miR-21-5p | Clin remission postind | Wilcoxon | 39 | median(success) - median(fail) | -0.0893 | 0.6685 | 0.9066 |
| Pooled | Baseline | miR-362-3p | Clin remission postind | Wilcoxon | 39 | median(success) - median(fail) | -0.3176 | 0.5858 | 0.9066 |
| Pooled | Baseline | miR-376c-3p | Clin remission postind | Wilcoxon | 39 | median(success) - median(fail) | -0.1918 | 0.907 | 0.907 |
| Pooled | Baseline | miR-424-5p | Clin remission postind | Wilcoxon | 39 | median(success) - median(fail) | -0.3958 | 0.4596 | 0.9066 |
| Pooled | Baseline | miR-106a-5p | FC 12m | Spearman | 38 | rho | 0.1323 | 0.4286 | 0.8572 |
| Pooled | Baseline | miR-146b-5p | FC 12m | Spearman | 38 | rho | -0.1388 | 0.4058 | 0.8572 |
| Pooled | Baseline | miR-21-5p | FC 12m | Spearman | 38 | rho | 0.0551 | 0.7426 | 0.8911 |
| Pooled | Baseline | miR-362-3p | FC 12m | Spearman | 38 | rho | -0.0049 | 0.9766 | 0.9766 |
| Pooled | Baseline | miR-376c-3p | FC 12m | Spearman | 38 | rho | 0.3338 | 0.0405 | 0.2431 |
| Pooled | Baseline | miR-424-5p | FC 12m | Spearman | 38 | rho | -0.0616 | 0.7131 | 0.8911 |
| Pooled | Baseline | miR-106a-5p | FC postind | Spearman | 39 | rho | -0.0428 | 0.7957 | 0.7957 |
| Pooled | Baseline | miR-146b-5p | FC postind | Spearman | 39 | rho | -0.0689 | 0.6766 | 0.7957 |
| Pooled | Baseline | miR-21-5p | FC postind | Spearman | 39 | rho | 0.0444 | 0.7882 | 0.7957 |
| Pooled | Baseline | miR-362-3p | FC postind | Spearman | 39 | rho | 0.1075 | 0.5147 | 0.7957 |
| Pooled | Baseline | miR-376c-3p | FC postind | Spearman | 39 | rho | 0.0915 | 0.5795 | 0.7957 |
| Pooled | Baseline | miR-424-5p | FC postind | Spearman | 39 | rho | 0.2849 | 0.0788 | 0.4726 |
| Pooled | Baseline | miR-106a-5p | FC remission 12m | Wilcoxon | 38 | median(success) - median(fail) | 0.1546 | 0.5032 | 0.6304 |
| Pooled | Baseline | miR-146b-5p | FC remission 12m | Wilcoxon | 38 | median(success) - median(fail) | 0.282 | 0.1265 | 0.4626 |
| Pooled | Baseline | miR-21-5p | FC remission 12m | Wilcoxon | 38 | median(success) - median(fail) | 0.0764 | 0.5253 | 0.6304 |
| Pooled | Baseline | miR-362-3p | FC remission 12m | Wilcoxon | 38 | median(success) - median(fail) | -0.0159 | 0.693 | 0.693 |
| Pooled | Baseline | miR-376c-3p | FC remission 12m | Wilcoxon | 38 | median(success) - median(fail) | -0.8806 | 0.1542 | 0.4626 |
| Pooled | Baseline | miR-424-5p | FC remission 12m | Wilcoxon | 38 | median(success) - median(fail) | 0.0205 | 0.3451 | 0.6304 |
| Pooled | Baseline | miR-106a-5p | FC remission postind | Wilcoxon | 39 | median(success) - median(fail) | -0.0503 | 0.6995 | 0.8394 |
| Pooled | Baseline | miR-146b-5p | FC remission postind | Wilcoxon | 39 | median(success) - median(fail) | -0.0056 | 0.6295 | 0.8394 |
| Pooled | Baseline | miR-21-5p | FC remission postind | Wilcoxon | 39 | median(success) - median(fail) | 0.1639 | 0.9487 | 0.9487 |
| Pooled | Baseline | miR-362-3p | FC remission postind | Wilcoxon | 39 | median(success) - median(fail) | -0.251 | 0.351 | 0.8394 |
| Pooled | Baseline | miR-376c-3p | FC remission postind | Wilcoxon | 39 | median(success) - median(fail) | -0.4185 | 0.6525 | 0.8394 |
| Pooled | Baseline | miR-424-5p | FC remission postind | Wilcoxon | 39 | median(success) - median(fail) | -0.4616 | 0.0824 | 0.4945 |
| Pooled | Baseline | miR-106a-5p | HBI 12m | Spearman | 35 | rho | 0.0668 | 0.703 | 0.8428 |
| Pooled | Baseline | miR-146b-5p | HBI 12m | Spearman | 35 | rho | 0.0557 | 0.7505 | 0.8428 |
| Pooled | Baseline | miR-21-5p | HBI 12m | Spearman | 35 | rho | 0.0511 | 0.7705 | 0.8428 |
| Pooled | Baseline | miR-362-3p | HBI 12m | Spearman | 35 | rho | 0.2086 | 0.2292 | 0.7587 |
| Pooled | Baseline | miR-376c-3p | HBI 12m | Spearman | 35 | rho | 0.0348 | 0.8428 | 0.8428 |
| Pooled | Baseline | miR-424-5p | HBI 12m | Spearman | 35 | rho | -0.1985 | 0.2529 | 0.7587 |
| Pooled | Baseline | miR-106a-5p | HBI postind | Spearman | 39 | rho | -0.1833 | 0.2641 | 0.5282 |
| Pooled | Baseline | miR-146b-5p | HBI postind | Spearman | 39 | rho | 0.2714 | 0.0946 | 0.5282 |
| Pooled | Baseline | miR-21-5p | HBI postind | Spearman | 39 | rho | 0.0404 | 0.8073 | 0.8073 |
| Pooled | Baseline | miR-362-3p | HBI postind | Spearman | 39 | rho | -0.1065 | 0.5188 | 0.7782 |
| Pooled | Baseline | miR-376c-3p | HBI postind | Spearman | 39 | rho | 0.2154 | 0.188 | 0.5282 |
| Pooled | Baseline | miR-424-5p | HBI postind | Spearman | 39 | rho | 0.054 | 0.744 | 0.8073 |
| Pooled | Baseline | miR-106a-5p | SES-CD 12m | Spearman | 30 | rho | 0.2817 | 0.1316 | 0.2631 |
| Pooled | Baseline | miR-146b-5p | SES-CD 12m | Spearman | 30 | rho | -0.1658 | 0.3811 | 0.5716 |
| Pooled | Baseline | miR-21-5p | SES-CD 12m | Spearman | 30 | rho | -0.2842 | 0.128 | 0.2631 |
| Pooled | Baseline | miR-362-3p | SES-CD 12m | Spearman | 30 | rho | -0.0551 | 0.7726 | 0.7726 |
| Pooled | Baseline | miR-376c-3p | SES-CD 12m | Spearman | 30 | rho | -0.1229 | 0.5176 | 0.6211 |
| Pooled | Baseline | miR-424-5p | SES-CD 12m | Spearman | 30 | rho | -0.3223 | 0.0824 | 0.2631 |
| Pooled | Baseline | miR-106a-5p | Treatment success | Wilcoxon | 32 | median(success) - median(fail) | 0.0779 | 0.7155 | 0.9541 |
| Pooled | Baseline | miR-146b-5p | Treatment success | Wilcoxon | 32 | median(success) - median(fail) | -0.1558 | 0.8932 | 0.9541 |
| Pooled | Baseline | miR-21-5p | Treatment success | Wilcoxon | 32 | median(success) - median(fail) | -0.1054 | 0.388 | 0.9541 |
| Pooled | Baseline | miR-362-3p | Treatment success | Wilcoxon | 32 | median(success) - median(fail) | -0.2398 | 0.687 | 0.9541 |
| Pooled | Baseline | miR-376c-3p | Treatment success | Wilcoxon | 32 | median(success) - median(fail) | -0.1855 | 0.9541 | 0.9541 |
| Pooled | Baseline | miR-424-5p | Treatment success | Wilcoxon | 32 | median(success) - median(fail) | 0.0021 | 0.9541 | 0.9541 |
| Pooled | Delta | miR-106a-5p | CRP 12m | Spearman | 37 | rho | 0.0328 | 0.8471 | 0.8471 |
| Pooled | Delta | miR-146b-5p | CRP 12m | Spearman | 37 | rho | 0.4201 | 0.0096 | 0.0578 |
| Pooled | Delta | miR-21-5p | CRP 12m | Spearman | 37 | rho | 0.1793 | 0.2882 | 0.3553 |
| Pooled | Delta | miR-362-3p | CRP 12m | Spearman | 35 | rho | 0.1996 | 0.2504 | 0.3553 |
| Pooled | Delta | miR-376c-3p | CRP 12m | Spearman | 37 | rho | 0.1765 | 0.2961 | 0.3553 |
| Pooled | Delta | miR-424-5p | CRP 12m | Spearman | 37 | rho | 0.2971 | 0.0741 | 0.2224 |
| Pooled | Delta | miR-106a-5p | CRP postind | Spearman | 39 | rho | 0.1916 | 0.2427 | 0.4129 |
| Pooled | Delta | miR-146b-5p | CRP postind | Spearman | 39 | rho | 0.3114 | 0.0536 | 0.198 |
| Pooled | Delta | miR-21-5p | CRP postind | Spearman | 39 | rho | 0.1327 | 0.4205 | 0.5046 |
| Pooled | Delta | miR-362-3p | CRP postind | Spearman | 37 | rho | 0.0972 | 0.5672 | 0.5672 |
| Pooled | Delta | miR-376c-3p | CRP postind | Spearman | 39 | rho | 0.1791 | 0.2753 | 0.4129 |
| Pooled | Delta | miR-424-5p | CRP postind | Spearman | 39 | rho | 0.2974 | 0.066 | 0.198 |
| Pooled | Delta | miR-106a-5p | Clin remission 12m | Wilcoxon | 35 | median(success) - median(fail) | 0.0205 | 0.5919 | 0.8161 |
| Pooled | Delta | miR-146b-5p | Clin remission 12m | Wilcoxon | 35 | median(success) - median(fail) | -0.2596 | 0.9015 | 0.9015 |
| Pooled | Delta | miR-21-5p | Clin remission 12m | Wilcoxon | 35 | median(success) - median(fail) | -0.2083 | 0.0762 | 0.4571 |
| Pooled | Delta | miR-362-3p | Clin remission 12m | Wilcoxon | 33 | median(success) - median(fail) | -0.104 | 0.3551 | 0.7102 |
| Pooled | Delta | miR-376c-3p | Clin remission 12m | Wilcoxon | 35 | median(success) - median(fail) | -0.3379 | 0.6801 | 0.8161 |
| Pooled | Delta | miR-424-5p | Clin remission 12m | Wilcoxon | 35 | median(success) - median(fail) | 0.4823 | 0.3223 | 0.7102 |
| Pooled | Delta | miR-106a-5p | Clin remission postind | Wilcoxon | 39 | median(success) - median(fail) | -0.0595 | 0.8763 | 0.907 |
| Pooled | Delta | miR-146b-5p | Clin remission postind | Wilcoxon | 39 | median(success) - median(fail) | 0.432 | 0.3305 | 0.907 |
| Pooled | Delta | miR-21-5p | Clin remission postind | Wilcoxon | 39 | median(success) - median(fail) | -0.0954 | 0.6685 | 0.907 |
| Pooled | Delta | miR-362-3p | Clin remission postind | Wilcoxon | 37 | median(success) - median(fail) | -0.2388 | 0.4583 | 0.907 |
| Pooled | Delta | miR-376c-3p | Clin remission postind | Wilcoxon | 39 | median(success) - median(fail) | -0.0715 | 0.6685 | 0.907 |
| Pooled | Delta | miR-424-5p | Clin remission postind | Wilcoxon | 39 | median(success) - median(fail) | 0.0547 | 0.907 | 0.907 |
| Pooled | Delta | miR-106a-5p | FC 12m | Spearman | 38 | rho | -0.1123 | 0.5019 | 0.7528 |
| Pooled | Delta | miR-146b-5p | FC 12m | Spearman | 38 | rho | 0.137 | 0.4122 | 0.7528 |
| Pooled | Delta | miR-21-5p | FC 12m | Spearman | 38 | rho | -0.0566 | 0.7357 | 0.8828 |
| Pooled | Delta | miR-362-3p | FC 12m | Spearman | 36 | rho | -0.1435 | 0.4038 | 0.7528 |
| Pooled | Delta | miR-376c-3p | FC 12m | Spearman | 38 | rho | -0.128 | 0.4438 | 0.7528 |
| Pooled | Delta | miR-424-5p | FC 12m | Spearman | 38 | rho | 0.0047 | 0.9776 | 0.9776 |
| Pooled | Delta | miR-106a-5p | FC postind | Spearman | 39 | rho | -0.1519 | 0.3561 | 0.5498 |
| Pooled | Delta | miR-146b-5p | FC postind | Spearman | 39 | rho | 0.1486 | 0.3665 | 0.5498 |
| Pooled | Delta | miR-21-5p | FC postind | Spearman | 39 | rho | -0.0856 | 0.6042 | 0.725 |
| Pooled | Delta | miR-362-3p | FC postind | Spearman | 37 | rho | 0.0017 | 0.9922 | 0.9922 |
| Pooled | Delta | miR-376c-3p | FC postind | Spearman | 39 | rho | -0.1645 | 0.3169 | 0.5498 |
| Pooled | Delta | miR-424-5p | FC postind | Spearman | 39 | rho | 0.3144 | 0.0513 | 0.3078 |
| Pooled | Delta | miR-106a-5p | FC remission 12m | Wilcoxon | 38 | median(success) - median(fail) | -0.019 | 0.3451 | 0.9316 |
| Pooled | Delta | miR-146b-5p | FC remission 12m | Wilcoxon | 38 | median(success) - median(fail) | -0.1544 | 0.7443 | 0.9316 |
| Pooled | Delta | miR-21-5p | FC remission 12m | Wilcoxon | 38 | median(success) - median(fail) | 0.0967 | 0.9316 | 0.9316 |
| Pooled | Delta | miR-362-3p | FC remission 12m | Wilcoxon | 36 | median(success) - median(fail) | -0.0201 | 0.7841 | 0.9316 |
| Pooled | Delta | miR-376c-3p | FC remission 12m | Wilcoxon | 38 | median(success) - median(fail) | -0.2879 | 0.5032 | 0.9316 |
| Pooled | Delta | miR-424-5p | FC remission 12m | Wilcoxon | 38 | median(success) - median(fail) | -0.015 | 0.9043 | 0.9316 |
| Pooled | Delta | miR-106a-5p | FC remission postind | Wilcoxon | 39 | median(success) - median(fail) | 0.1782 | 0.4792 | 0.9584 |
| Pooled | Delta | miR-146b-5p | FC remission postind | Wilcoxon | 39 | median(success) - median(fail) | -0.1891 | 0.8219 | 0.9743 |
| Pooled | Delta | miR-21-5p | FC remission postind | Wilcoxon | 39 | median(success) - median(fail) | 0.0028 | 0.9743 | 0.9743 |
| Pooled | Delta | miR-362-3p | FC remission postind | Wilcoxon | 37 | median(success) - median(fail) | -0.0976 | 0.9119 | 0.9743 |
| Pooled | Delta | miR-376c-3p | FC remission postind | Wilcoxon | 39 | median(success) - median(fail) | 0.3202 | 0.4402 | 0.9584 |
| Pooled | Delta | miR-424-5p | FC remission postind | Wilcoxon | 39 | median(success) - median(fail) | -0.0664 | 0.2885 | 0.9584 |
| Pooled | Delta | miR-106a-5p | HBI 12m | Spearman | 35 | rho | -0.1165 | 0.5051 | 0.8645 |
| Pooled | Delta | miR-146b-5p | HBI 12m | Spearman | 35 | rho | -0.031 | 0.8595 | 0.8645 |
| Pooled | Delta | miR-21-5p | HBI 12m | Spearman | 35 | rho | 0.1247 | 0.4754 | 0.8645 |
| Pooled | Delta | miR-362-3p | HBI 12m | Spearman | 33 | rho | 0.0309 | 0.8645 | 0.8645 |
| Pooled | Delta | miR-376c-3p | HBI 12m | Spearman | 35 | rho | -0.0553 | 0.7523 | 0.8645 |
| Pooled | Delta | miR-424-5p | HBI 12m | Spearman | 35 | rho | 0.1299 | 0.4571 | 0.8645 |
| Pooled | Delta | miR-106a-5p | HBI postind | Spearman | 39 | rho | -0.0154 | 0.9258 | 0.9258 |
| Pooled | Delta | miR-146b-5p | HBI postind | Spearman | 39 | rho | -0.3712 | 0.02 | 0.1199 |
| Pooled | Delta | miR-21-5p | HBI postind | Spearman | 39 | rho | -0.0357 | 0.8292 | 0.9258 |
| Pooled | Delta | miR-362-3p | HBI postind | Spearman | 37 | rho | 0.216 | 0.1991 | 0.3983 |
| Pooled | Delta | miR-376c-3p | HBI postind | Spearman | 39 | rho | -0.3075 | 0.0569 | 0.1707 |
| Pooled | Delta | miR-424-5p | HBI postind | Spearman | 39 | rho | -0.1352 | 0.412 | 0.618 |
| Pooled | Delta | miR-106a-5p | SES-CD 12m | Spearman | 30 | rho | -0.3468 | 0.0605 | 0.2919 |
| Pooled | Delta | miR-146b-5p | SES-CD 12m | Spearman | 30 | rho | 0.1439 | 0.448 | 0.5376 |
| Pooled | Delta | miR-21-5p | SES-CD 12m | Spearman | 30 | rho | 0.1757 | 0.3531 | 0.5297 |
| Pooled | Delta | miR-362-3p | SES-CD 12m | Spearman | 28 | rho | -0.1131 | 0.5667 | 0.5667 |
| Pooled | Delta | miR-376c-3p | SES-CD 12m | Spearman | 30 | rho | 0.2051 | 0.2768 | 0.5297 |
| Pooled | Delta | miR-424-5p | SES-CD 12m | Spearman | 30 | rho | 0.3084 | 0.0973 | 0.2919 |
| Pooled | Delta | miR-106a-5p | Treatment success | Wilcoxon | 32 | median(success) - median(fail) | -0.0309 | 0.687 | 0.9828 |
| Pooled | Delta | miR-146b-5p | Treatment success | Wilcoxon | 32 | median(success) - median(fail) | -0.452 | 0.1502 | 0.9012 |
| Pooled | Delta | miR-21-5p | Treatment success | Wilcoxon | 32 | median(success) - median(fail) | -0.1355 | 0.659 | 0.9828 |
| Pooled | Delta | miR-362-3p | Treatment success | Wilcoxon | 30 | median(success) - median(fail) | 0.0723 | 0.9828 | 0.9828 |
| Pooled | Delta | miR-376c-3p | Treatment success | Wilcoxon | 32 | median(success) - median(fail) | -0.2256 | 0.9541 | 0.9828 |
| Pooled | Delta | miR-424-5p | Treatment success | Wilcoxon | 32 | median(success) - median(fail) | 0.1312 | 0.8629 | 0.9828 |
| Pooled | Post-induction | miR-106a-5p | CRP 12m | Spearman | 37 | rho | -0.1975 | 0.2414 | 0.5474 |
| Pooled | Post-induction | miR-146b-5p | CRP 12m | Spearman | 37 | rho | 0.2434 | 0.1466 | 0.5474 |
| Pooled | Post-induction | miR-21-5p | CRP 12m | Spearman | 37 | rho | 0.1277 | 0.4514 | 0.5474 |
| Pooled | Post-induction | miR-362-3p | CRP 12m | Spearman | 35 | rho | 0.1301 | 0.4562 | 0.5474 |
| Pooled | Post-induction | miR-376c-3p | CRP 12m | Spearman | 37 | rho | 0.011 | 0.9486 | 0.9486 |
| Pooled | Post-induction | miR-424-5p | CRP 12m | Spearman | 37 | rho | 0.1394 | 0.4107 | 0.5474 |
| Pooled | Post-induction | miR-106a-5p | CRP postind | Spearman | 39 | rho | -0.1823 | 0.2667 | 0.5335 |
| Pooled | Post-induction | miR-146b-5p | CRP postind | Spearman | 39 | rho | 0.0561 | 0.7346 | 0.8961 |
| Pooled | Post-induction | miR-21-5p | CRP postind | Spearman | 39 | rho | 0.1827 | 0.2656 | 0.5335 |
| Pooled | Post-induction | miR-362-3p | CRP postind | Spearman | 37 | rho | 0.0235 | 0.89 | 0.8961 |
| Pooled | Post-induction | miR-376c-3p | CRP postind | Spearman | 39 | rho | -0.0216 | 0.8961 | 0.8961 |
| Pooled | Post-induction | miR-424-5p | CRP postind | Spearman | 39 | rho | 0.4399 | 0.0051 | 0.0305 |
| Pooled | Post-induction | miR-106a-5p | Clin remission 12m | Wilcoxon | 35 | median(success) - median(fail) | 0.0766 | 0.9343 | 0.9343 |
| Pooled | Post-induction | miR-146b-5p | Clin remission 12m | Wilcoxon | 35 | median(success) - median(fail) | 0.3956 | 0.8366 | 0.9343 |
| Pooled | Post-induction | miR-21-5p | Clin remission 12m | Wilcoxon | 35 | median(success) - median(fail) | -0.0934 | 0.2317 | 0.9158 |
| Pooled | Post-induction | miR-362-3p | Clin remission 12m | Wilcoxon | 33 | median(success) - median(fail) | -0.2088 | 0.4541 | 0.9158 |
| Pooled | Post-induction | miR-376c-3p | Clin remission 12m | Wilcoxon | 35 | median(success) - median(fail) | -0.0937 | 0.9343 | 0.9343 |
| Pooled | Post-induction | miR-424-5p | Clin remission 12m | Wilcoxon | 35 | median(success) - median(fail) | -0.0532 | 0.4579 | 0.9158 |
| Pooled | Post-induction | miR-106a-5p | Clin remission postind | Wilcoxon | 39 | median(success) - median(fail) | 0.3398 | 0.0356 | 0.2133 |
| Pooled | Post-induction | miR-146b-5p | Clin remission postind | Wilcoxon | 39 | median(success) - median(fail) | 0.4252 | 0.5081 | 0.6098 |
| Pooled | Post-induction | miR-21-5p | Clin remission postind | Wilcoxon | 39 | median(success) - median(fail) | -0.0076 | 0.8763 | 0.8763 |
| Pooled | Post-induction | miR-362-3p | Clin remission postind | Wilcoxon | 37 | median(success) - median(fail) | -0.6691 | 0.0835 | 0.2506 |
| Pooled | Post-induction | miR-376c-3p | Clin remission postind | Wilcoxon | 39 | median(success) - median(fail) | 0.5591 | 0.3918 | 0.5877 |
| Pooled | Post-induction | miR-424-5p | Clin remission postind | Wilcoxon | 39 | median(success) - median(fail) | -0.2403 | 0.2276 | 0.4551 |
| Pooled | Post-induction | miR-106a-5p | FC 12m | Spearman | 38 | rho | 0.0499 | 0.7659 | 0.7754 |
| Pooled | Post-induction | miR-146b-5p | FC 12m | Spearman | 38 | rho | 0.0514 | 0.7595 | 0.7754 |
| Pooled | Post-induction | miR-21-5p | FC 12m | Spearman | 38 | rho | -0.0554 | 0.7411 | 0.7754 |
| Pooled | Post-induction | miR-362-3p | FC 12m | Spearman | 36 | rho | -0.1954 | 0.2535 | 0.7754 |
| Pooled | Post-induction | miR-376c-3p | FC 12m | Spearman | 38 | rho | 0.1216 | 0.4669 | 0.7754 |
| Pooled | Post-induction | miR-424-5p | FC 12m | Spearman | 38 | rho | -0.0478 | 0.7754 | 0.7754 |
| Pooled | Post-induction | miR-106a-5p | FC postind | Spearman | 39 | rho | -0.3522 | 0.0279 | 0.0836 |
| Pooled | Post-induction | miR-146b-5p | FC postind | Spearman | 39 | rho | 0.1128 | 0.4942 | 0.8311 |
| Pooled | Post-induction | miR-21-5p | FC postind | Spearman | 39 | rho | 0.0818 | 0.6206 | 0.8311 |
| Pooled | Post-induction | miR-362-3p | FC postind | Spearman | 37 | rho | 0.0672 | 0.6926 | 0.8311 |
| Pooled | Post-induction | miR-376c-3p | FC postind | Spearman | 39 | rho | -0.0299 | 0.8568 | 0.8568 |
| Pooled | Post-induction | miR-424-5p | FC postind | Spearman | 39 | rho | 0.5225 | 0.0006 | 0.0039 |
| Pooled | Post-induction | miR-106a-5p | FC remission 12m | Wilcoxon | 38 | median(success) - median(fail) | 0.016 | 0.3813 | 0.7547 |
| Pooled | Post-induction | miR-146b-5p | FC remission 12m | Wilcoxon | 38 | median(success) - median(fail) | 0.4177 | 0.4398 | 0.7547 |
| Pooled | Post-induction | miR-21-5p | FC remission 12m | Wilcoxon | 38 | median(success) - median(fail) | 0.0283 | 0.5032 | 0.7547 |
| Pooled | Post-induction | miR-362-3p | FC remission 12m | Wilcoxon | 36 | median(success) - median(fail) | -0.0951 | 0.7013 | 0.8415 |
| Pooled | Post-induction | miR-376c-3p | FC remission 12m | Wilcoxon | 38 | median(success) - median(fail) | -0.0475 | 0.9863 | 0.9863 |
| Pooled | Post-induction | miR-424-5p | FC remission 12m | Wilcoxon | 38 | median(success) - median(fail) | 0.444 | 0.3278 | 0.7547 |
| Pooled | Post-induction | miR-106a-5p | FC remission postind | Wilcoxon | 39 | median(success) - median(fail) | 0.2428 | 0.0577 | 0.1732 |
| Pooled | Post-induction | miR-146b-5p | FC remission postind | Wilcoxon | 39 | median(success) - median(fail) | 0.0222 | 0.5201 | 0.9231 |
| Pooled | Post-induction | miR-21-5p | FC remission postind | Wilcoxon | 39 | median(success) - median(fail) | 0.0794 | 0.847 | 0.9231 |
| Pooled | Post-induction | miR-362-3p | FC remission postind | Wilcoxon | 37 | median(success) - median(fail) | -0.0705 | 0.658 | 0.9231 |
| Pooled | Post-induction | miR-376c-3p | FC remission postind | Wilcoxon | 39 | median(success) - median(fail) | 0.0422 | 0.9231 | 0.9231 |
| Pooled | Post-induction | miR-424-5p | FC remission postind | Wilcoxon | 39 | median(success) - median(fail) | -0.6025 | 0.0092 | 0.0551 |
| Pooled | Post-induction | miR-106a-5p | HBI 12m | Spearman | 35 | rho | -0.0573 | 0.7436 | 0.9543 |
| Pooled | Post-induction | miR-146b-5p | HBI 12m | Spearman | 35 | rho | 0.0677 | 0.6993 | 0.9543 |
| Pooled | Post-induction | miR-21-5p | HBI 12m | Spearman | 35 | rho | 0.2166 | 0.2113 | 0.9543 |
| Pooled | Post-induction | miR-362-3p | HBI 12m | Spearman | 33 | rho | 0.0734 | 0.6846 | 0.9543 |
| Pooled | Post-induction | miR-376c-3p | HBI 12m | Spearman | 35 | rho | -0.0101 | 0.9543 | 0.9543 |
| Pooled | Post-induction | miR-424-5p | HBI 12m | Spearman | 35 | rho | -0.0142 | 0.9354 | 0.9543 |
| Pooled | Post-induction | miR-106a-5p | HBI postind | Spearman | 39 | rho | -0.2897 | 0.0737 | 0.2551 |
| Pooled | Post-induction | miR-146b-5p | HBI postind | Spearman | 39 | rho | -0.1685 | 0.3052 | 0.6105 |
| Pooled | Post-induction | miR-21-5p | HBI postind | Spearman | 39 | rho | 0.0615 | 0.7101 | 0.982 |
| Pooled | Post-induction | miR-362-3p | HBI postind | Spearman | 37 | rho | 0.287 | 0.085 | 0.2551 |
| Pooled | Post-induction | miR-376c-3p | HBI postind | Spearman | 39 | rho | -0.0366 | 0.8248 | 0.982 |
| Pooled | Post-induction | miR-424-5p | HBI postind | Spearman | 39 | rho | -0.0037 | 0.982 | 0.982 |
| Pooled | Post-induction | miR-106a-5p | SES-CD 12m | Spearman | 30 | rho | -0.1373 | 0.4694 | 0.9388 |
| Pooled | Post-induction | miR-146b-5p | SES-CD 12m | Spearman | 30 | rho | -0.0139 | 0.9417 | 0.9417 |
| Pooled | Post-induction | miR-21-5p | SES-CD 12m | Spearman | 30 | rho | -0.1574 | 0.4062 | 0.9388 |
| Pooled | Post-induction | miR-362-3p | SES-CD 12m | Spearman | 28 | rho | -0.3449 | 0.0723 | 0.4336 |
| Pooled | Post-induction | miR-376c-3p | SES-CD 12m | Spearman | 30 | rho | -0.0384 | 0.8404 | 0.9417 |
| Pooled | Post-induction | miR-424-5p | SES-CD 12m | Spearman | 30 | rho | 0.0578 | 0.7616 | 0.9417 |
| Pooled | Post-induction | miR-106a-5p | Treatment success | Wilcoxon | 32 | median(success) - median(fail) | -0.071 | 0.9236 | 0.9236 |
| Pooled | Post-induction | miR-146b-5p | Treatment success | Wilcoxon | 32 | median(success) - median(fail) | -0.2209 | 0.1614 | 0.4841 |
| Pooled | Post-induction | miR-21-5p | Treatment success | Wilcoxon | 32 | median(success) - median(fail) | -0.1978 | 0.1296 | 0.4841 |
| Pooled | Post-induction | miR-362-3p | Treatment success | Wilcoxon | 30 | median(success) - median(fail) | 0.1756 | 0.5047 | 0.9236 |
| Pooled | Post-induction | miR-376c-3p | Treatment success | Wilcoxon | 32 | median(success) - median(fail) | 0.0475 | 0.8031 | 0.9236 |
| Pooled | Post-induction | miR-424-5p | Treatment success | Wilcoxon | 32 | median(success) - median(fail) | 0.0614 | 0.7735 | 0.9236 |
| VDZ | Baseline | miR-106a-5p | CRP 12m | Spearman | 18 | rho | -0.107 | 0.6726 | 0.7337 |
| VDZ | Baseline | miR-146b-5p | CRP 12m | Spearman | 18 | rho | -0.454 | 0.0584 | 0.3504 |
| VDZ | Baseline | miR-21-5p | CRP 12m | Spearman | 18 | rho | -0.3553 | 0.1479 | 0.4437 |
| VDZ | Baseline | miR-362-3p | CRP 12m | Spearman | 18 | rho | 0.0862 | 0.7337 | 0.7337 |
| VDZ | Baseline | miR-376c-3p | CRP 12m | Spearman | 18 | rho | -0.2629 | 0.292 | 0.5839 |
| VDZ | Baseline | miR-424-5p | CRP 12m | Spearman | 18 | rho | -0.1101 | 0.6635 | 0.7337 |
| VDZ | Baseline | miR-106a-5p | CRP postind | Spearman | 19 | rho | -0.6299 | 0.0038 | 0.0231 |
| VDZ | Baseline | miR-146b-5p | CRP postind | Spearman | 19 | rho | -0.3158 | 0.1877 | 0.456 |
| VDZ | Baseline | miR-21-5p | CRP postind | Spearman | 19 | rho | -0.0759 | 0.7575 | 0.909 |
| VDZ | Baseline | miR-362-3p | CRP postind | Spearman | 19 | rho | 0.1862 | 0.4454 | 0.6682 |
| VDZ | Baseline | miR-376c-3p | CRP postind | Spearman | 19 | rho | 0.0159 | 0.9486 | 0.9486 |
| VDZ | Baseline | miR-424-5p | CRP postind | Spearman | 19 | rho | 0.2903 | 0.228 | 0.456 |
| VDZ | Baseline | miR-106a-5p | Clin remission 12m | Wilcoxon | 16 | median(success) - median(fail) | 0.1859 | 0.4593 | 0.7366 |
| VDZ | Baseline | miR-146b-5p | Clin remission 12m | Wilcoxon | 16 | median(success) - median(fail) | -0.0257 | 0.6377 | 0.7366 |
| VDZ | Baseline | miR-21-5p | Clin remission 12m | Wilcoxon | 16 | median(success) - median(fail) | 0.1337 | 0.7366 | 0.7366 |
| VDZ | Baseline | miR-362-3p | Clin remission 12m | Wilcoxon | 16 | median(success) - median(fail) | 0.8629 | 0.4593 | 0.7366 |
| VDZ | Baseline | miR-376c-3p | Clin remission 12m | Wilcoxon | 16 | median(success) - median(fail) | -0.7008 | 0.6377 | 0.7366 |
| VDZ | Baseline | miR-424-5p | Clin remission 12m | Wilcoxon | 16 | median(success) - median(fail) | 0.6338 | 0.4593 | 0.7366 |
| VDZ | Baseline | miR-106a-5p | Clin remission postind | Wilcoxon | 19 | median(success) - median(fail) | 0.513 | 0.0139 | 0.0834 |
| VDZ | Baseline | miR-146b-5p | Clin remission postind | Wilcoxon | 19 | median(success) - median(fail) | 0.0883 | 0.7373 | 0.8231 |
| VDZ | Baseline | miR-21-5p | Clin remission postind | Wilcoxon | 19 | median(success) - median(fail) | 0.9942 | 0.1175 | 0.3526 |
| VDZ | Baseline | miR-362-3p | Clin remission postind | Wilcoxon | 19 | median(success) - median(fail) | -0.2957 | 0.8231 | 0.8231 |
| VDZ | Baseline | miR-376c-3p | Clin remission postind | Wilcoxon | 19 | median(success) - median(fail) | -0.9668 | 0.5023 | 0.8231 |
| VDZ | Baseline | miR-424-5p | Clin remission postind | Wilcoxon | 19 | median(success) - median(fail) | -0.7053 | 0.7373 | 0.8231 |
| VDZ | Baseline | miR-106a-5p | FC 12m | Spearman | 19 | rho | 0.1125 | 0.6466 | 0.9699 |
| VDZ | Baseline | miR-146b-5p | FC 12m | Spearman | 19 | rho | -0.0264 | 0.9147 | 0.9829 |
| VDZ | Baseline | miR-21-5p | FC 12m | Spearman | 19 | rho | 0.2601 | 0.2822 | 0.8465 |
| VDZ | Baseline | miR-362-3p | FC 12m | Spearman | 19 | rho | -0.1336 | 0.5857 | 0.9699 |
| VDZ | Baseline | miR-376c-3p | FC 12m | Spearman | 19 | rho | 0.2917 | 0.2255 | 0.8465 |
| VDZ | Baseline | miR-424-5p | FC 12m | Spearman | 19 | rho | 0.0053 | 0.9829 | 0.9829 |
| VDZ | Baseline | miR-106a-5p | FC postind | Spearman | 19 | rho | -0.2684 | 0.2665 | 0.702 |
| VDZ | Baseline | miR-146b-5p | FC postind | Spearman | 19 | rho | -0.0825 | 0.7372 | 0.8846 |
| VDZ | Baseline | miR-21-5p | FC postind | Spearman | 19 | rho | 0.1947 | 0.4243 | 0.702 |
| VDZ | Baseline | miR-362-3p | FC postind | Spearman | 19 | rho | 0.1772 | 0.468 | 0.702 |
| VDZ | Baseline | miR-376c-3p | FC postind | Spearman | 19 | rho | 0.0211 | 0.9318 | 0.9318 |
| VDZ | Baseline | miR-424-5p | FC postind | Spearman | 19 | rho | 0.1789 | 0.4636 | 0.702 |
| VDZ | Baseline | miR-106a-5p | FC remission 12m | Wilcoxon | 19 | median(success) - median(fail) | 0.1484 | 0.911 | 0.911 |
| VDZ | Baseline | miR-146b-5p | FC remission 12m | Wilcoxon | 19 | median(success) - median(fail) | 0.0763 | 0.3711 | 0.7422 |
| VDZ | Baseline | miR-21-5p | FC remission 12m | Wilcoxon | 19 | median(success) - median(fail) | -0.0018 | 0.911 | 0.911 |
| VDZ | Baseline | miR-362-3p | FC remission 12m | Wilcoxon | 19 | median(success) - median(fail) | 0.639 | 0.0573 | 0.3441 |
| VDZ | Baseline | miR-376c-3p | FC remission 12m | Wilcoxon | 19 | median(success) - median(fail) | -0.8083 | 0.5023 | 0.7535 |
| VDZ | Baseline | miR-424-5p | FC remission 12m | Wilcoxon | 19 | median(success) - median(fail) | 0.9428 | 0.3711 | 0.7422 |
| VDZ | Baseline | miR-106a-5p | FC remission postind | Wilcoxon | 19 | median(success) - median(fail) | 0.2341 | 0.2301 | 0.3452 |
| VDZ | Baseline | miR-146b-5p | FC remission postind | Wilcoxon | 19 | median(success) - median(fail) | -0.1584 | 0.8415 | 0.8415 |
| VDZ | Baseline | miR-21-5p | FC remission postind | Wilcoxon | 19 | median(success) - median(fail) | -0.3242 | 0.1615 | 0.323 |
| VDZ | Baseline | miR-362-3p | FC remission postind | Wilcoxon | 19 | median(success) - median(fail) | -0.734 | 0.0574 | 0.323 |
| VDZ | Baseline | miR-376c-3p | FC remission postind | Wilcoxon | 19 | median(success) - median(fail) | -0.7373 | 0.8415 | 0.8415 |
| VDZ | Baseline | miR-424-5p | FC remission postind | Wilcoxon | 19 | median(success) - median(fail) | -1.0823 | 0.1096 | 0.323 |
| VDZ | Baseline | miR-106a-5p | HBI 12m | Spearman | 16 | rho | -0.0121 | 0.9644 | 0.9644 |
| VDZ | Baseline | miR-146b-5p | HBI 12m | Spearman | 16 | rho | 0.1456 | 0.5905 | 0.8857 |
| VDZ | Baseline | miR-21-5p | HBI 12m | Spearman | 16 | rho | 0.1487 | 0.5827 | 0.8857 |
| VDZ | Baseline | miR-362-3p | HBI 12m | Spearman | 16 | rho | 0.0546 | 0.8408 | 0.9644 |
| VDZ | Baseline | miR-376c-3p | HBI 12m | Spearman | 16 | rho | 0.2321 | 0.387 | 0.8857 |
| VDZ | Baseline | miR-424-5p | HBI 12m | Spearman | 16 | rho | -0.1517 | 0.5749 | 0.8857 |
| VDZ | Baseline | miR-106a-5p | HBI postind | Spearman | 19 | rho | -0.3212 | 0.18 | 0.7996 |
| VDZ | Baseline | miR-146b-5p | HBI postind | Spearman | 19 | rho | 0.0789 | 0.748 | 0.8976 |
| VDZ | Baseline | miR-21-5p | HBI postind | Spearman | 19 | rho | -0.1857 | 0.4466 | 0.7996 |
| VDZ | Baseline | miR-362-3p | HBI postind | Spearman | 19 | rho | -0.1525 | 0.5331 | 0.7996 |
| VDZ | Baseline | miR-376c-3p | HBI postind | Spearman | 19 | rho | 0.2404 | 0.3215 | 0.7996 |
| VDZ | Baseline | miR-424-5p | HBI postind | Spearman | 19 | rho | 0.017 | 0.9448 | 0.9448 |
| VDZ | Baseline | miR-106a-5p | SES-CD 12m | Spearman | 12 | rho | -0.018 | 0.9558 | 0.9558 |
| VDZ | Baseline | miR-146b-5p | SES-CD 12m | Spearman | 12 | rho | 0.194 | 0.5458 | 0.8166 |
| VDZ | Baseline | miR-21-5p | SES-CD 12m | Spearman | 12 | rho | -0.194 | 0.5458 | 0.8166 |
| VDZ | Baseline | miR-362-3p | SES-CD 12m | Spearman | 12 | rho | -0.2047 | 0.5233 | 0.8166 |
| VDZ | Baseline | miR-376c-3p | SES-CD 12m | Spearman | 12 | rho | -0.1329 | 0.6805 | 0.8166 |
| VDZ | Baseline | miR-424-5p | SES-CD 12m | Spearman | 12 | rho | -0.2694 | 0.3971 | 0.8166 |
| VDZ | Baseline | miR-106a-5p | Treatment success | Wilcoxon | 17 | median(success) - median(fail) | 0.0578 | 0.5637 | 0.6764 |
| VDZ | Baseline | miR-146b-5p | Treatment success | Wilcoxon | 17 | median(success) - median(fail) | -0.3327 | 0.1779 | 0.6764 |
| VDZ | Baseline | miR-21-5p | Treatment success | Wilcoxon | 17 | median(success) - median(fail) | -0.5301 | 0.3865 | 0.6764 |
| VDZ | Baseline | miR-362-3p | Treatment success | Wilcoxon | 17 | median(success) - median(fail) | 0.0976 | 0.7003 | 0.7003 |
| VDZ | Baseline | miR-376c-3p | Treatment success | Wilcoxon | 17 | median(success) - median(fail) | -1.1563 | 0.4414 | 0.6764 |
| VDZ | Baseline | miR-424-5p | Treatment success | Wilcoxon | 17 | median(success) - median(fail) | -0.0598 | 0.5006 | 0.6764 |
| VDZ | Delta | miR-106a-5p | CRP 12m | Spearman | 18 | rho | -0.426 | 0.078 | 0.1675 |
| VDZ | Delta | miR-146b-5p | CRP 12m | Spearman | 18 | rho | 0.4904 | 0.0388 | 0.1675 |
| VDZ | Delta | miR-21-5p | CRP 12m | Spearman | 18 | rho | 0.3086 | 0.2128 | 0.2554 |
| VDZ | Delta | miR-362-3p | CRP 12m | Spearman | 16 | rho | 0.346 | 0.1893 | 0.2554 |
| VDZ | Delta | miR-376c-3p | CRP 12m | Spearman | 18 | rho | 0.1538 | 0.5424 | 0.5424 |
| VDZ | Delta | miR-424-5p | CRP 12m | Spearman | 18 | rho | 0.4187 | 0.0837 | 0.1675 |
| VDZ | Delta | miR-106a-5p | CRP postind | Spearman | 19 | rho | 0.3238 | 0.1763 | 0.8093 |
| VDZ | Delta | miR-146b-5p | CRP postind | Spearman | 19 | rho | 0.1853 | 0.4476 | 0.8093 |
| VDZ | Delta | miR-21-5p | CRP postind | Spearman | 19 | rho | 0.1279 | 0.6017 | 0.8093 |
| VDZ | Delta | miR-362-3p | CRP postind | Spearman | 17 | rho | 0.1099 | 0.6744 | 0.8093 |
| VDZ | Delta | miR-376c-3p | CRP postind | Spearman | 19 | rho | 0.0512 | 0.8352 | 0.8352 |
| VDZ | Delta | miR-424-5p | CRP postind | Spearman | 19 | rho | 0.1332 | 0.5867 | 0.8093 |
| VDZ | Delta | miR-106a-5p | Clin remission 12m | Wilcoxon | 16 | median(success) - median(fail) | 0.0741 | 0.6377 | 0.6377 |
| VDZ | Delta | miR-146b-5p | Clin remission 12m | Wilcoxon | 16 | median(success) - median(fail) | -0.4987 | 0.2012 | 0.4024 |
| VDZ | Delta | miR-21-5p | Clin remission 12m | Wilcoxon | 16 | median(success) - median(fail) | -0.158 | 0.2012 | 0.4024 |
| VDZ | Delta | miR-362-3p | Clin remission 12m | Wilcoxon | 14 | median(success) - median(fail) | -0.6151 | 0.1391 | 0.4024 |
| VDZ | Delta | miR-376c-3p | Clin remission 12m | Wilcoxon | 16 | median(success) - median(fail) | -0.3277 | 0.313 | 0.4695 |
| VDZ | Delta | miR-424-5p | Clin remission 12m | Wilcoxon | 16 | median(success) - median(fail) | -0.1163 | 0.5449 | 0.6377 |
| VDZ | Delta | miR-106a-5p | Clin remission postind | Wilcoxon | 19 | median(success) - median(fail) | -0.1483 | 0.7373 | 0.8848 |
| VDZ | Delta | miR-146b-5p | Clin remission postind | Wilcoxon | 19 | median(success) - median(fail) | 0.0282 | 0.7373 | 0.8848 |
| VDZ | Delta | miR-21-5p | Clin remission postind | Wilcoxon | 19 | median(success) - median(fail) | -0.1382 | 0.1797 | 0.8848 |
| VDZ | Delta | miR-362-3p | Clin remission postind | Wilcoxon | 17 | median(success) - median(fail) | -0.2659 | 0.5287 | 0.8848 |
| VDZ | Delta | miR-376c-3p | Clin remission postind | Wilcoxon | 19 | median(success) - median(fail) | -0.1837 | 0.911 | 0.911 |
| VDZ | Delta | miR-424-5p | Clin remission postind | Wilcoxon | 19 | median(success) - median(fail) | -0.4404 | 0.4338 | 0.8848 |
| VDZ | Delta | miR-106a-5p | FC 12m | Spearman | 19 | rho | -0.174 | 0.4762 | 0.5715 |
| VDZ | Delta | miR-146b-5p | FC 12m | Spearman | 19 | rho | 0.0984 | 0.6885 | 0.6885 |
| VDZ | Delta | miR-21-5p | FC 12m | Spearman | 19 | rho | -0.2566 | 0.289 | 0.5715 |
| VDZ | Delta | miR-362-3p | FC 12m | Spearman | 17 | rho | -0.1941 | 0.4554 | 0.5715 |
| VDZ | Delta | miR-376c-3p | FC 12m | Spearman | 19 | rho | -0.188 | 0.4407 | 0.5715 |
| VDZ | Delta | miR-424-5p | FC 12m | Spearman | 19 | rho | -0.2882 | 0.2314 | 0.5715 |
| VDZ | Delta | miR-106a-5p | FC postind | Spearman | 19 | rho | -0.1246 | 0.6114 | 0.9171 |
| VDZ | Delta | miR-146b-5p | FC postind | Spearman | 19 | rho | 0.2263 | 0.3515 | 0.9171 |
| VDZ | Delta | miR-21-5p | FC postind | Spearman | 19 | rho | 0.0228 | 0.9262 | 0.9943 |
| VDZ | Delta | miR-362-3p | FC postind | Spearman | 17 | rho | 0.1544 | 0.554 | 0.9171 |
| VDZ | Delta | miR-376c-3p | FC postind | Spearman | 19 | rho | 0.0018 | 0.9943 | 0.9943 |
| VDZ | Delta | miR-424-5p | FC postind | Spearman | 19 | rho | 0.1386 | 0.5715 | 0.9171 |
| VDZ | Delta | miR-106a-5p | FC remission 12m | Wilcoxon | 19 | median(success) - median(fail) | -0.0063 | 0.5762 | 0.8642 |
| VDZ | Delta | miR-146b-5p | FC remission 12m | Wilcoxon | 19 | median(success) - median(fail) | -0.7373 | 0.5023 | 0.8642 |
| VDZ | Delta | miR-21-5p | FC remission 12m | Wilcoxon | 19 | median(success) - median(fail) | -0.0058 | 0.911 | 0.911 |
| VDZ | Delta | miR-362-3p | FC remission 12m | Wilcoxon | 17 | median(success) - median(fail) | -0.1892 | 0.8011 | 0.911 |
| VDZ | Delta | miR-376c-3p | FC remission 12m | Wilcoxon | 19 | median(success) - median(fail) | -0.1241 | 0.5762 | 0.8642 |
| VDZ | Delta | miR-424-5p | FC remission 12m | Wilcoxon | 19 | median(success) - median(fail) | 0.0197 | 0.5023 | 0.8642 |
| VDZ | Delta | miR-106a-5p | FC remission postind | Wilcoxon | 19 | median(success) - median(fail) | -0.0091 | 0.7642 | 0.917 |
| VDZ | Delta | miR-146b-5p | FC remission postind | Wilcoxon | 19 | median(success) - median(fail) | -0.0847 | 0.3681 | 0.917 |
| VDZ | Delta | miR-21-5p | FC remission postind | Wilcoxon | 19 | median(success) - median(fail) | 0.0028 | 0.6892 | 0.917 |
| VDZ | Delta | miR-362-3p | FC remission postind | Wilcoxon | 17 | median(success) - median(fail) | 1.8373 | 0.233 | 0.917 |
| VDZ | Delta | miR-376c-3p | FC remission postind | Wilcoxon | 19 | median(success) - median(fail) | -0.6012 | 1 | 1 |
| VDZ | Delta | miR-424-5p | FC remission postind | Wilcoxon | 19 | median(success) - median(fail) | 0.2535 | 0.4839 | 0.917 |
| VDZ | Delta | miR-106a-5p | HBI 12m | Spearman | 16 | rho | -0.3292 | 0.2131 | 0.4262 |
| VDZ | Delta | miR-146b-5p | HBI 12m | Spearman | 16 | rho | 0.4066 | 0.1181 | 0.3634 |
| VDZ | Delta | miR-21-5p | HBI 12m | Spearman | 16 | rho | 0.1411 | 0.6022 | 0.7227 |
| VDZ | Delta | miR-362-3p | HBI 12m | Spearman | 14 | rho | 0.1867 | 0.5228 | 0.7227 |
| VDZ | Delta | miR-376c-3p | HBI 12m | Spearman | 16 | rho | 0.0319 | 0.9068 | 0.9068 |
| VDZ | Delta | miR-424-5p | HBI 12m | Spearman | 16 | rho | 0.4035 | 0.1211 | 0.3634 |
| VDZ | Delta | miR-106a-5p | HBI postind | Spearman | 19 | rho | 0.0664 | 0.7871 | 0.7871 |
| VDZ | Delta | miR-146b-5p | HBI postind | Spearman | 19 | rho | -0.3104 | 0.1959 | 0.6769 |
| VDZ | Delta | miR-21-5p | HBI postind | Spearman | 19 | rho | 0.2323 | 0.3385 | 0.6769 |
| VDZ | Delta | miR-362-3p | HBI postind | Spearman | 17 | rho | 0.2804 | 0.2756 | 0.6769 |
| VDZ | Delta | miR-376c-3p | HBI postind | Spearman | 19 | rho | -0.1328 | 0.5879 | 0.7645 |
| VDZ | Delta | miR-424-5p | HBI postind | Spearman | 19 | rho | -0.1157 | 0.6371 | 0.7645 |
| VDZ | Delta | miR-106a-5p | SES-CD 12m | Spearman | 12 | rho | -0.1509 | 0.6398 | 0.9206 |
| VDZ | Delta | miR-146b-5p | SES-CD 12m | Spearman | 12 | rho | -0.0395 | 0.903 | 0.9206 |
| VDZ | Delta | miR-21-5p | SES-CD 12m | Spearman | 12 | rho | -0.176 | 0.5843 | 0.9206 |
| VDZ | Delta | miR-362-3p | SES-CD 12m | Spearman | 10 | rho | -0.0556 | 0.8788 | 0.9206 |
| VDZ | Delta | miR-376c-3p | SES-CD 12m | Spearman | 12 | rho | 0.0611 | 0.8505 | 0.9206 |
| VDZ | Delta | miR-424-5p | SES-CD 12m | Spearman | 12 | rho | 0.0323 | 0.9206 | 0.9206 |
| VDZ | Delta | miR-106a-5p | Treatment success | Wilcoxon | 17 | median(success) - median(fail) | -0.1707 | 0.7728 | 0.8474 |
| VDZ | Delta | miR-146b-5p | Treatment success | Wilcoxon | 17 | median(success) - median(fail) | -0.3858 | 0.2482 | 0.6718 |
| VDZ | Delta | miR-21-5p | Treatment success | Wilcoxon | 17 | median(success) - median(fail) | 0.0731 | 0.7003 | 0.8474 |
| VDZ | Delta | miR-362-3p | Treatment success | Wilcoxon | 15 | median(success) - median(fail) | -0.2659 | 0.2472 | 0.6718 |
| VDZ | Delta | miR-376c-3p | Treatment success | Wilcoxon | 17 | median(success) - median(fail) | 0.7496 | 0.3359 | 0.6718 |
| VDZ | Delta | miR-424-5p | Treatment success | Wilcoxon | 17 | median(success) - median(fail) | 0.1108 | 0.8474 | 0.8474 |
| VDZ | Post-induction | miR-106a-5p | CRP 12m | Spearman | 18 | rho | -0.4291 | 0.0756 | 0.4535 |
| VDZ | Post-induction | miR-146b-5p | CRP 12m | Spearman | 18 | rho | 0.1849 | 0.4625 | 0.9717 |
| VDZ | Post-induction | miR-21-5p | CRP 12m | Spearman | 18 | rho | 0.0603 | 0.8122 | 0.9935 |
| VDZ | Post-induction | miR-362-3p | CRP 12m | Spearman | 16 | rho | 0.052 | 0.8484 | 0.9935 |
| VDZ | Post-induction | miR-376c-3p | CRP 12m | Spearman | 18 | rho | -0.0021 | 0.9935 | 0.9935 |
| VDZ | Post-induction | miR-424-5p | CRP 12m | Spearman | 18 | rho | 0.1756 | 0.4859 | 0.9717 |
| VDZ | Post-induction | miR-106a-5p | CRP postind | Spearman | 19 | rho | -0.2479 | 0.3061 | 0.8432 |
| VDZ | Post-induction | miR-146b-5p | CRP postind | Spearman | 19 | rho | 0.0609 | 0.8045 | 0.8432 |
| VDZ | Post-induction | miR-21-5p | CRP postind | Spearman | 19 | rho | 0.1923 | 0.4302 | 0.8432 |
| VDZ | Post-induction | miR-362-3p | CRP postind | Spearman | 17 | rho | 0.0519 | 0.8432 | 0.8432 |
| VDZ | Post-induction | miR-376c-3p | CRP postind | Spearman | 19 | rho | 0.0918 | 0.7087 | 0.8432 |
| VDZ | Post-induction | miR-424-5p | CRP postind | Spearman | 19 | rho | 0.4279 | 0.0676 | 0.4057 |
| VDZ | Post-induction | miR-106a-5p | Clin remission 12m | Wilcoxon | 16 | median(success) - median(fail) | 0.2847 | 0.5449 | 0.7029 |
| VDZ | Post-induction | miR-146b-5p | Clin remission 12m | Wilcoxon | 16 | median(success) - median(fail) | -0.8424 | 0.0511 | 0.2779 |
| VDZ | Post-induction | miR-21-5p | Clin remission 12m | Wilcoxon | 16 | median(success) - median(fail) | -0.4675 | 0.0926 | 0.2779 |
| VDZ | Post-induction | miR-362-3p | Clin remission 12m | Wilcoxon | 14 | median(success) - median(fail) | -0.336 | 0.5858 | 0.7029 |
| VDZ | Post-induction | miR-376c-3p | Clin remission 12m | Wilcoxon | 16 | median(success) - median(fail) | -0.4224 | 0.3819 | 0.7029 |
| VDZ | Post-induction | miR-424-5p | Clin remission 12m | Wilcoxon | 16 | median(success) - median(fail) | -0.1028 | 0.9464 | 0.9464 |
| VDZ | Post-induction | miR-106a-5p | Clin remission postind | Wilcoxon | 19 | median(success) - median(fail) | 0.3155 | 0.0442 | 0.265 |
| VDZ | Post-induction | miR-146b-5p | Clin remission postind | Wilcoxon | 19 | median(success) - median(fail) | 0.5696 | 0.7373 | 0.7373 |
| VDZ | Post-induction | miR-21-5p | Clin remission postind | Wilcoxon | 19 | median(success) - median(fail) | 0.1684 | 0.6547 | 0.7373 |
| VDZ | Post-induction | miR-362-3p | Clin remission postind | Wilcoxon | 17 | median(success) - median(fail) | -0.7306 | 0.3135 | 0.6286 |
| VDZ | Post-induction | miR-376c-3p | Clin remission postind | Wilcoxon | 19 | median(success) - median(fail) | 0.0157 | 0.6547 | 0.7373 |
| VDZ | Post-induction | miR-424-5p | Clin remission postind | Wilcoxon | 19 | median(success) - median(fail) | -0.1666 | 0.3143 | 0.6286 |
| VDZ | Post-induction | miR-106a-5p | FC 12m | Spearman | 19 | rho | 0.0246 | 0.9204 | 0.9204 |
| VDZ | Post-induction | miR-146b-5p | FC 12m | Spearman | 19 | rho | 0.1986 | 0.415 | 0.6226 |
| VDZ | Post-induction | miR-21-5p | FC 12m | Spearman | 19 | rho | -0.1002 | 0.6832 | 0.8199 |
| VDZ | Post-induction | miR-362-3p | FC 12m | Spearman | 17 | rho | -0.4054 | 0.1064 | 0.6226 |
| VDZ | Post-induction | miR-376c-3p | FC 12m | Spearman | 19 | rho | 0.2232 | 0.3583 | 0.6226 |
| VDZ | Post-induction | miR-424-5p | FC 12m | Spearman | 19 | rho | -0.283 | 0.2405 | 0.6226 |
| VDZ | Post-induction | miR-106a-5p | FC postind | Spearman | 19 | rho | -0.4053 | 0.0852 | 0.1764 |
| VDZ | Post-induction | miR-146b-5p | FC postind | Spearman | 19 | rho | 0.3491 | 0.1429 | 0.2144 |
| VDZ | Post-induction | miR-21-5p | FC postind | Spearman | 19 | rho | 0.4018 | 0.0882 | 0.1764 |
| VDZ | Post-induction | miR-362-3p | FC postind | Spearman | 17 | rho | 0.0539 | 0.8372 | 0.8372 |
| VDZ | Post-induction | miR-376c-3p | FC postind | Spearman | 19 | rho | 0.2684 | 0.2665 | 0.3198 |
| VDZ | Post-induction | miR-424-5p | FC postind | Spearman | 19 | rho | 0.4105 | 0.0808 | 0.1764 |
| VDZ | Post-induction | miR-106a-5p | FC remission 12m | Wilcoxon | 19 | median(success) - median(fail) | -0.3794 | 0.2636 | 0.5271 |
| VDZ | Post-induction | miR-146b-5p | FC remission 12m | Wilcoxon | 19 | median(success) - median(fail) | -0.301 | 1 | 1 |
| VDZ | Post-induction | miR-21-5p | FC remission 12m | Wilcoxon | 19 | median(success) - median(fail) | 0.0122 | 0.5762 | 0.8642 |
| VDZ | Post-induction | miR-362-3p | FC remission 12m | Wilcoxon | 17 | median(success) - median(fail) | 0.5635 | 0.2568 | 0.5271 |
| VDZ | Post-induction | miR-376c-3p | FC remission 12m | Wilcoxon | 19 | median(success) - median(fail) | -0.1291 | 0.8231 | 0.9877 |
| VDZ | Post-induction | miR-424-5p | FC remission 12m | Wilcoxon | 19 | median(success) - median(fail) | 0.6049 | 0.0442 | 0.265 |
| VDZ | Post-induction | miR-106a-5p | FC remission postind | Wilcoxon | 19 | median(success) - median(fail) | 0.2433 | 0.3173 | 0.3808 |
| VDZ | Post-induction | miR-146b-5p | FC remission postind | Wilcoxon | 19 | median(success) - median(fail) | -0.8033 | 0.0574 | 0.2004 |
| VDZ | Post-induction | miR-21-5p | FC remission postind | Wilcoxon | 19 | median(success) - median(fail) | -0.4261 | 0.0891 | 0.2004 |
| VDZ | Post-induction | miR-362-3p | FC remission postind | Wilcoxon | 17 | median(success) - median(fail) | 0.7221 | 0.7656 | 0.7656 |
| VDZ | Post-induction | miR-376c-3p | FC remission postind | Wilcoxon | 19 | median(success) - median(fail) | -0.6471 | 0.1336 | 0.2004 |
| VDZ | Post-induction | miR-424-5p | FC remission postind | Wilcoxon | 19 | median(success) - median(fail) | -0.5656 | 0.1336 | 0.2004 |
| VDZ | Post-induction | miR-106a-5p | HBI 12m | Spearman | 16 | rho | -0.2063 | 0.4433 | 0.5825 |
| VDZ | Post-induction | miR-146b-5p | HBI 12m | Spearman | 16 | rho | 0.5643 | 0.0228 | 0.1366 |
| VDZ | Post-induction | miR-21-5p | HBI 12m | Spearman | 16 | rho | 0.3823 | 0.1439 | 0.4318 |
| VDZ | Post-induction | miR-362-3p | HBI 12m | Spearman | 14 | rho | 0.1138 | 0.6984 | 0.6984 |
| VDZ | Post-induction | miR-376c-3p | HBI 12m | Spearman | 16 | rho | 0.2776 | 0.2979 | 0.5825 |
| VDZ | Post-induction | miR-424-5p | HBI 12m | Spearman | 16 | rho | 0.1881 | 0.4854 | 0.5825 |
| VDZ | Post-induction | miR-106a-5p | HBI postind | Spearman | 19 | rho | -0.2135 | 0.3801 | 0.7055 |
| VDZ | Post-induction | miR-146b-5p | HBI postind | Spearman | 19 | rho | -0.244 | 0.3141 | 0.7055 |
| VDZ | Post-induction | miR-21-5p | HBI postind | Spearman | 19 | rho | 0.1328 | 0.5879 | 0.7055 |
| VDZ | Post-induction | miR-362-3p | HBI postind | Spearman | 17 | rho | 0.3345 | 0.1894 | 0.7055 |
| VDZ | Post-induction | miR-376c-3p | HBI postind | Spearman | 19 | rho | 0.0215 | 0.9303 | 0.9303 |
| VDZ | Post-induction | miR-424-5p | HBI postind | Spearman | 19 | rho | 0.1337 | 0.5854 | 0.7055 |
| VDZ | Post-induction | miR-106a-5p | SES-CD 12m | Spearman | 12 | rho | 0.1509 | 0.6398 | 0.8592 |
| VDZ | Post-induction | miR-146b-5p | SES-CD 12m | Spearman | 12 | rho | 0.0611 | 0.8505 | 0.8592 |
| VDZ | Post-induction | miR-21-5p | SES-CD 12m | Spearman | 12 | rho | -0.4382 | 0.1542 | 0.3475 |
| VDZ | Post-induction | miR-362-3p | SES-CD 12m | Spearman | 10 | rho | -0.6112 | 0.0605 | 0.3475 |
| VDZ | Post-induction | miR-376c-3p | SES-CD 12m | Spearman | 12 | rho | -0.0575 | 0.8592 | 0.8592 |
| VDZ | Post-induction | miR-424-5p | SES-CD 12m | Spearman | 12 | rho | -0.4203 | 0.1737 | 0.3475 |
| VDZ | Post-induction | miR-106a-5p | Treatment success | Wilcoxon | 17 | median(success) - median(fail) | 0.0882 | 0.7003 | 0.8404 |
| VDZ | Post-induction | miR-146b-5p | Treatment success | Wilcoxon | 17 | median(success) - median(fail) | -0.6295 | 0.0209 | 0.1255 |
| VDZ | Post-induction | miR-21-5p | Treatment success | Wilcoxon | 17 | median(success) - median(fail) | -0.3146 | 0.3359 | 0.8404 |
| VDZ | Post-induction | miR-362-3p | Treatment success | Wilcoxon | 15 | median(success) - median(fail) | 0.1639 | 1 | 1 |
| VDZ | Post-induction | miR-376c-3p | Treatment success | Wilcoxon | 17 | median(success) - median(fail) | -0.455 | 0.5637 | 0.8404 |
| VDZ | Post-induction | miR-424-5p | Treatment success | Wilcoxon | 17 | median(success) - median(fail) | 0.271 | 0.5006 | 0.8404 |

**Supplementary Table S6.** Spearman rank correlations between each baseline serum miRNA and baseline SES-CD as a continuous variable (n = 30 patients with available baseline endoscopy), with Benjamini–Hochberg FDR-corrected q-values. All q-values were 0.93, indicating that no panel miRNA tracked endoscopic activity at baseline. Cited in Section 3.4.

| **mirna** | **n** | **rho** | **p** | **q_BH** |
| --- | --- | --- | --- | --- |
| miR-21-5p | 30 | -0.048884 | 0.797545 | 0.934686 |
| miR-146b-5p | 30 | 0.015625 | 0.934686 | 0.934686 |
| miR-106a-5p | 30 | -0.142188 | 0.45354 | 0.934686 |
| miR-362-3p | 30 | 0.060045 | 0.752618 | 0.934686 |
| miR-376c-3p | 30 | -0.251787 | 0.179519 | 0.934686 |
| miR-424-5p | 30 | 0.072322 | 0.704102 | 0.934686 |

**Supplementary Table S7.** Spearman rank correlations of post-induction miR-424-5p with concurrent faecal calprotectin and concurrent CRP, re-derived under three normalisation strategies: the primary strategy used throughout the manuscript (geometric mean of miR-93-5p and miR-425-5p) and each reference miRNA singly. Cited in Section 3.4.

| **Normalisation_strategy** | **rho_miR424_x_FC_postind** | **p_miR424_x_FC_postind** | **rho_miR424_x_CRP_postind** | **p_miR424_x_CRP_postind** |
| --- | --- | --- | --- | --- |
| Geometric mean of miR-93-5p and miR-425-5p (manuscript primary) | 0.523 | 0.001 | 0.440 | 0.005 |
| miR-93-5p alone | 0.514 | 0.001 | 0.471 | 0.003 |
| miR-425-5p alone | 0.509 | 0.001 | 0.407 | 0.010 |

**Supplementary Table S8.** Leave-one-out single-observation sensitivity analysis for the four pooled-cohort headline correlations. Each patient was removed in turn and the Spearman rho recomputed; the table reports the full-sample rho with its p-value together with the minimum and maximum rho across the leave-one-out iterations, and the number of iterations in which the direction (n_dir_changes) or the statistical significance (n_sig_changes) of the association changed. Cited in Section 3.4.

| **label** | **n** | **full_rho** | **full_p** | **rho_min_LOO** | **rho_max_LOO** | **n_dir_changes** | **n_sig_changes** |
| --- | --- | --- | --- | --- | --- | --- | --- |
| post-induction miR-424-5p × FC postind | 39 | 0.522501 | 0.000644 | 0.495376 | 0.556012 | 0 | 0 |
| post-induction miR-424-5p × CRP postind | 39 | 0.439891 | 0.005076 | 0.400409 | 0.493812 | 0 | 0 |
| baseline miR-146b-5p × CRP postind | 39 | -0.385657 | 0.015321 | -0.468118 | -0.346038 | 0 | 0 |
| baseline miR-106a-5p × CRP postind | 39 | -0.406147 | 0.010302 | -0.5039 | -0.365231 | 0 | 0 |

**Supplementary Table S9.** Partial Spearman correlations of post-induction miR-424-5p with concurrent faecal calprotectin (FC postind) and concurrent CRP (CRP postind), adjusted one variable at a time for baseline HBI, SES-CD, CRP and faecal calprotectin, adjusted for treatment arm, and adjusted in a single multivariable model additionally including prior anti-TNF exposure and disease duration, with 95% confidence intervals and n for each model. All confidence intervals exclude zero. Cited in Section 3.4.

| **outcome** | **adjusted_for** | **rho** | **CI_low** | **CI_high** | **n** |
| --- | --- | --- | --- | --- | --- |
| FC postind | HBI baseline | 0.515 | 0.23 | 0.72 | 39 |
| FC postind | SES-CD baseline | 0.572 | 0.26 | 0.78 | 30 |
| FC postind | CRP baseline | 0.522 | 0.24 | 0.72 | 39 |
| FC postind | FC baseline | 0.517 | 0.24 | 0.72 | 39 |
| FC postind | treatment arm | 0.527 | 0.25 | 0.72 | 39 |
| FC postind | treatment arm + prior anti-TNF + disease duration | 0.516 | 0.23 | 0.72 | 39 |
| CRP postind | HBI baseline | 0.428 | 0.13 | 0.66 | 39 |
| CRP postind | SES-CD baseline | 0.474 | 0.13 | 0.72 | 30 |
| CRP postind | CRP baseline | 0.445 | 0.15 | 0.67 | 39 |
| CRP postind | FC baseline | 0.453 | 0.16 | 0.67 | 39 |
| CRP postind | treatment arm | 0.5 | 0.21 | 0.71 | 39 |
| CRP postind | treatment arm + prior anti-TNF + disease duration | 0.512 | 0.22 | 0.72 | 39 |

**Supplementary Table S10.** Treatment success at last follow-up re-analysed under a continuation-only definition, in which success was therapy continuation and failure was discontinuation due to inefficacy or loss of response only, excluding the patients who stopped therapy because of sustained remission and those who discontinued for unrelated reasons. The between-arm pattern is essentially identical to that of the primary composite outcome. Cited in Section 3.5.

| **Definition** | **n_INFX** | **n_INFX_success** | **n_VDZ** | **n_VDZ_success** | **Risk_diff_INFX_minus_VDZ** | **Odds_ratio** | **Fisher_p** |
| --- | --- | --- | --- | --- | --- | --- | --- |
| Treatment success = therapy continued; failure = discontinuation due to inefficacy or loss of response only (excluding stopped due to sustained remission and stopped for other reasons) | 13 | 9 | 17 | 8 | 0.222 | 2.45 | 0.283 |

**Supplementary Table S11.** Comprehensive Montreal B3 (penetrating) versus non-B3 (B1+B2, non-penetrating) phenotype contrast for all six panel miRNAs at all three predictor timepoints (baseline v1, post-induction v4 and treatment-induced change Δ = v4 − v1; 18 contrasts), with Mann–Whitney p-values and Benjamini–Hochberg FDR-corrected q-values across the full scan. The treatment-induced change in miR-106a-5p was the only contrast with nominal p < 0.05 and did not survive FDR correction across the 18 contrasts. Cited in Section 3.7.

| **timepoint** | **mirna** | **n_B3** | **n_nonB3** | **median_B3** | **median_nonB3** | **median_diff_B3_minus_nonB3** | **Wilcoxon_p** | **q_BH** |
| --- | --- | --- | --- | --- | --- | --- | --- | --- |
| Baseline (v1) | miR-21-5p | 19 | 20 | -0.2838 | -0.4517 | 0.168 | 0.227 | 0.5836 |
| Baseline (v1) | miR-146b-5p | 19 | 20 | -0.4395 | -0.7272 | 0.2877 | 0.3993 | 0.6794 |
| Baseline (v1) | miR-106a-5p | 19 | 20 | -0.0921 | 0.0319 | -0.1239 | 0.3118 | 0.6794 |
| Baseline (v1) | miR-362-3p | 19 | 20 | 0.2778 | 0.609 | -0.3313 | 0.4152 | 0.6794 |
| Baseline (v1) | miR-376c-3p | 19 | 20 | -0.3191 | -1.1891 | 0.87 | 0.227 | 0.5836 |
| Baseline (v1) | miR-424-5p | 19 | 20 | 0.0325 | -0.2018 | 0.2343 | 0.1601 | 0.5836 |
| Post-induction (v4) | miR-21-5p | 19 | 20 | -0.1731 | -0.3357 | 0.1626 | 0.3686 | 0.6794 |
| Post-induction (v4) | miR-146b-5p | 19 | 20 | -0.2778 | -0.4542 | 0.1764 | 0.8221 | 0.9249 |
| Post-induction (v4) | miR-106a-5p | 19 | 20 | 0.1389 | -0.0506 | 0.1895 | 0.0598 | 0.5379 |
| Post-induction (v4) | miR-362-3p | 18 | 19 | 0.2013 | 0.2907 | -0.0894 | 0.9758 | 0.9758 |
| Post-induction (v4) | miR-376c-3p | 19 | 20 | -0.6922 | -0.7344 | 0.0422 | 0.5365 | 0.7428 |
| Post-induction (v4) | miR-424-5p | 19 | 20 | -0.4585 | -0.3972 | -0.0613 | 0.6734 | 0.8314 |
| Delta (v4 - v1) | miR-21-5p | 19 | 20 | 0.1106 | 0.1048 | 0.0058 | 0.5365 | 0.7428 |
| Delta (v4 - v1) | miR-146b-5p | 19 | 20 | 0.4143 | 0.1392 | 0.2751 | 0.9552 | 0.9758 |
| Delta (v4 - v1) | miR-106a-5p | 19 | 20 | 0.2875 | -0.0617 | 0.3492 | 0.0124 | 0.2231 |
| Delta (v4 - v1) | miR-362-3p | 18 | 19 | 0.0234 | 0.0742 | -0.0508 | 0.6928 | 0.8314 |
| Delta (v4 - v1) | miR-376c-3p | 19 | 20 | 0.0673 | 0.6567 | -0.5894 | 0.1866 | 0.5836 |
| Delta (v4 - v1) | miR-424-5p | 19 | 20 | -0.3056 | -0.0758 | -0.2298 | 0.1601 | 0.5836 |
