# Supplementary material for: Circulating microRNAs as biomarkers of disease activity and treatment response in Crohn’s disease patients receiving infliximab or vedolizumab: a longitudinal cohort study
Source: Front Med (Lausanne). 2026 Aug 13;13:1923232. doi: 10.3389/fmed.2026.1923232 (PMC13518191; doi:10.3389/fmed.2026.1923232)
Supplement: Supplementary file 2 [file Table_2.docx]

**Supplementary Material**

**Supplementary Figures**

**Supplementary Figure S1.** Post-induction serum miR-424-5p (log₂ FC) plotted against concurrent (A) faecal calprotectin (μg/g, log scale) and (B) C-reactive protein (mg/L) in 39 CD patients at the post-induction timepoint, coloured by drug arm. Black lines show ordinary-least-squares fits; statistics are Spearman correlations on the original scale. Headline values: panel A — ρ = +0.52, p = 6.4 × 10⁻⁴, q = 0.004, n = 39; panel B — ρ = +0.44, p = 5.1 × 10⁻³, q = 0.030, n = 39. The associations were stronger within the INFX subgroup (FC ρ = +0.62, q = 0.020; CRP ρ = +0.63, q = 0.017; both n = 20) and remained directionally consistent in VDZ. Robustness to severity adjustment, leave-one-out removal, and single-reference normalisation is shown in Supplementary Figure S4 and the Supplementary_Sensitivity_Analyses supplement.

**Supplementary Figure S2.** Comprehensive forest plot of every FDR-significant F4 finding across pooled, INFX-only, and VDZ-only analyses. Pooled findings (reddish-purple): the four pooled associations listed in Figure 2. INFX subgroup (blue): post-induction miR-424-5p × concurrent FC (ρ = +0.62, q = 0.020, n = 20) and × concurrent CRP (ρ = +0.63, q = 0.017, n = 20). VDZ subgroup (orange): baseline miR-106a-5p × post-induction CRP (ρ = −0.63, q = 0.023, n = 19). Subgroup signals are exploratory and have wider confidence intervals than pooled estimates; the VDZ miR-106a-5p signal attenuates substantially after severity adjustment (Supplementary Figure S4) and is therefore not interpreted as a confirmed drug-specific predictor.

**Supplementary Figure S3.** Heatmap of Spearman rank correlations (ρ) between each of the six panel miRNAs and concurrent or subsequent biochemical inflammation markers (faecal calprotectin and C-reactive protein) in the pooled CD cohort. Rows are the six panel miRNAs; columns are six pre-specified predictor-outcome pairings linking baseline (v1) or post-induction (v4) miRNA values with FC and CRP at post-induction and 12 months. Cell colour indicates the magnitude and direction of the Spearman correlation; numerical values are overlaid on each cell. Asterisks (*) denote correlations that survived Benjamini-Hochberg FDR correction within the corresponding F4 cell (q < 0.05). The figure shows that miR-424-5p is the only panel miRNA with strong concurrent state-marker correlations (post-ind × post-ind row), while baseline miR-146b-5p and miR-106a-5p emerge as predictive baseline signals for post-induction CRP. The complete F4 family results (including INFX and VDZ subgroups and additional outcomes) are reported in Supplementary Table S3.

**Supplementary Figure S4.** Severity-adjusted Spearman ρ for the four pooled-cohort headline associations and the two VDZ subgroup miR-106a-5p associations. Each panel shows the unadjusted ρ (grey) and the partial ρ after adjusting for each of HBI baseline, SES-CD baseline, CRP baseline, FC baseline, or all four severity covariates jointly. Pooled findings (top four panels): all four headline correlations remain significant under every adjustment (smallest adjusted ρ = +0.43 for miR-424-5p × CRP under HBI adjustment, p = 0.007; largest = +0.57 for miR-424-5p × FC under SES-CD adjustment, p = 0.001). VDZ subgroup (bottom two panels): both miR-106a-5p associations attenuate substantially after SES-CD adjustment (clinical remission: ρ = +0.58 → +0.23, p = 0.45; CRP postind: ρ = −0.63 → −0.22, p = 0.47), indicating substantial confounding by baseline endoscopic severity. Adjusted-ALL estimate uses all four severity covariates simultaneously and is based on n = 30 (pooled) or n = 14 (VDZ) due to listwise deletion.

**Supplementary Figure S5.** Predictors of 12-month endoscopic activity (SES-CD at 12 months) in 30 patients with available 12-month endoscopic data. (A) Post-induction serum miR-424-5p (log₂ FC) versus 12-month SES-CD: Spearman ρ = +0.06, p = 0.76, n = 30 (null association). (B) Baseline faecal calprotectin (μg/g, log scale) versus 12-month SES-CD: Spearman ρ = +0.29, p = 0.12, n = 30 (null association). Black lines are OLS fits; points coloured by drug arm. Both predictors are null, confirming that miR-424-5p (like FC) is best characterised as a concurrent state monitor rather than a long-term prognostic marker.

**Supplementary Figure S6.** Baseline serum miRNA levels stratified by responder status, for two clinically meaningful outcomes. Top row: post-induction biochemical remission (faecal calprotectin < 250 μg/g) in the pooled cohort (n = 39; 29 responders, 10 non-responders). Bottom row: 12-month endoscopic remission (SES-CD < 3) in patients with available 12-month endoscopy (n = 30; 18 responders, 12 non-responders). Each column corresponds to one of the six panel miRNAs. Boxes show median and IQR with individual values overlaid; dashed line indicates the HC reference. Mann–Whitney p-values are reported within each panel. No contrast survives FDR correction across the 24 explored comparisons in this analysis; the two nominal signals (miR-106a-5p p = 0.048 and miR-424-5p p = 0.043, both for 12-month endoscopic remission) are presented as hypothesis-generating, not as confirmed baseline predictors.

**Supplementary Figure S7.** Receiver operating characteristic curves for the VDZ-arm post-induction predictors of treatment success at last follow-up: miR-146b-5p, AUC = 0.79 (95 % CI 0.53–1.00), n = 17 (8 success, 9 failure).

**Supplementary Figure S8.** Leave-one-out cross-validated AUCs for classifying clinically active disease (defined by HBI ≥ 5 alone; faecal calprotectin excluded from the label definition to avoid tautological circularity) across 78 pooled patient-timepoint observations (44 active, 34 inactive). Models include single biomarkers (FC, CRP, each of six panel miRNAs) and multi-marker combinations.

**Supplementary Figure S9.** Exploratory phenotype-stratified analysis (post-hoc): treatment-induced change in serum miR-106a-5p (Δ = v4 − v1, log₂ FC) by Montreal CD behaviour, B1+B2 (non-penetrating, n = 20) versus B3 (penetrating, n = 19). Patients with B3 disease showed an upregulation of miR-106a-5p over the induction period (median Δ = +0.29) whereas non-penetrating patients showed no net change (median Δ = −0.06). Mean difference in Δ between B3 and non-B3 = +0.314 log₂ FC; permutation-based p = 0.019 (50,000 label shuffles); partial r = +0.37 after adjustment for treatment success (permutation p = 0.042). This is presented as a single pre-declared exploratory contrast (section 3.7) and not as a confirmatory finding; it requires independent validation.
